# Fragment-Based Lead Generation of 5-Phenyl-1*H*-pyrazole-3-carboxamide Derivatives as Leads for Potent Factor Xia Inhibitors

**DOI:** 10.3390/molecules23082002

**Published:** 2018-08-10

**Authors:** Qunchao Wei, Zhichao Zheng, Shijun Zhang, Xuemin Zheng, Fancui Meng, Jing Yuan, Yongnan Xu, Changjiang Huang

**Affiliations:** 1 School of Pharmaceutical Engineering, Shenyang Pharmaceutical University, Shenyang 110016, China; wei_qunchao@126.com; 2 Tianjin Key Laboratory of Molecular Design and Drug Discovery, Tianjin Institute of Pharmaceutical Research, Tianjin 300193, China; zhengzc@tjipr.com (Z.Z.); zhangsj@tjipr.com (S.Z.); zhengxm@tjipr.com (X.Z.); mengfc@tjipr.com (F.M.); yuanj@tjipr.com (J.Y.)

**Keywords:** thrombosis, coagulation factors, FXIa inhibitors, docking stimulation, computer-aided drug design

## Abstract

FXIa is suggested as a major target for anticoagulant drug discovery because of reduced risk of bleeding. In this paper, we defined 5-phenyl-1*H*-pyrazole-3-carboxylic acid derivatives as privileged fragments for FXIa inhibitors’ lead discovery. After replacing the (*E*)-3-(5-chloro-2-(1*H*-tetrazol-1-yl)phenyl)acrylamide moiety in compound **3** with 5-(3-chlorophenyl)-1*H*-pyrazole-3-carboxamide, we traveled from FXIa inhibitor **3** to a scaffold that fused the privileged fragments into a pharmacophore for FXIa inhibitors. Subsequently, we synthesized and assessed the FXIa inhibitory potency of a series of 5-phenyl-1*H*-pyrazole-3-carboxamide derivatives with different P1, P1′ and P2′moiety. Finally, the SAR of them was systematically investigated to afford the lead compound **7za** (FXIa Ki = 90.37 nM, 1.5× aPTT in rabbit plasma = 43.33 μM) which exhibited good in vitro inhibitory potency against FXIa and excellent in vitro coagulation activities. Furthermore, the binding mode of **7za** with FXIa was studied and the results suggest that the 2-methylcyclopropanecarboxamide group of **7za** makes 2 direct hydrogen bonds with Tyr58B and Thr35 in the FXIa backbone, making **7za** binds to FXIa in a highly efficient manner.

## 1. Introduction

Cardiovascular (CV) disease continues to be the leading cause of death worldwide [1]. Thrombosis is the common underlying pathology of cardiovascular diseases and anticoagulants are the mainstay to prevent and/or treat thrombosis [2]. In clinical use, anticoagulants include antithrombin activators (heparins including unfractionated heparin, low molecular weight heparins and fondaparinux), vitamin K antagonists (coumarins such as warfarin), direct inhibitors of thrombin (hirudins, argatroban and dabigatran etexilate) and oral direct FXa inhibitors (rivaroxaban, apixaban, edoxaban and betrixaban) [3]. Although these agents possess high efficacy and relatively low cost to benefit ratio, they stillremain be associated with the life-threatening side effect of internal bleeding [4,5]. Therefore, despite the progresses made in past few years, there is also an urgent clinical need for developing new anticoagulants to prevent and/or treat thromboembolic diseases without the risk of bleeding or with low bleeding risk.

Generally, it is known from coagulation cascade that proteins in the intrinsic pathway are more important for the amplification phase of coagulation, whereas those belonging to the extrinsic and common coagulation pathways are more involved in the initiation and propagation phases. Current anticoagulants used for treating thrombosis mainly target two key serine proteases, thrombin and factor Xa (FXa), and they both belong to the common pathway of the coagulation cascade. Meanwhile, it has been postulated that selective inhibition of intrinsic coagulation factors could provide antithrombotic benefits with low bleeding risk because this will keep the other pathways of coagulation intact for hemostasis [6,7,8]. It’s shown by epidemiological and clinical studies that the inhibition of Factor XIa (FXIa) which belongs to the intrinsic pathway of the coagulation cascade has emerged as an excellent way to achieve anticoagulation without significant effects on hemostasis [9]. Human FXI deficiency (hemophilia C) was first described as a mild to moderate bleeding disorder [10]. However, these affected patients rarely suffer from spontaneous bleeding episodes. Furthermore, epidemiologic studies showed an increased risk of thrombosis in subjects with elevated FXI levels and some protection from thrombosis in subjects with reduced FXI levels [11,12]. Furthermore, the open-label, parallel-group Phase II study (NCT01713361) showed that reducing FXI levels specifically by an antisense oligonucleotide in patients undergoing elective knee arthroplasty was an effective method for postoperative venous thromboembolism prevention and appeared to be safe with respect to the risk of bleeding [13]. In addition, the Phase I study (NCT03197779) showed that the small molecule FXIa inhibitor BMS-962212 had good enough tolerability, pharmacokinetics and pharmacodynamics properties suitable for investigational use as an acute antithrombotic agent in Japanese or non-Japanese subjects [14,15]. In summary, FXIa is suggested as a major target for anticoagulant drug discovery because of reduced risk of bleeding [7].

**Figure 1:**
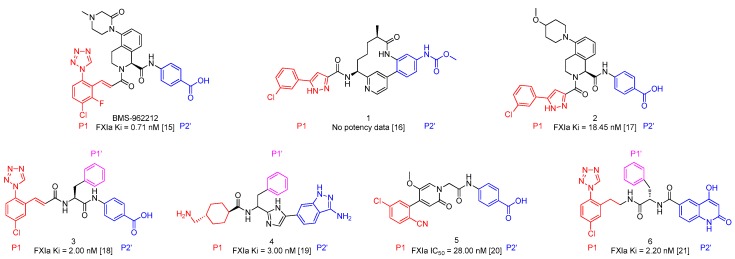
The representative FXIa inhibitors.

In recent years, lots of small molecule FXIa inhibitors were reported, including compounds **1**–**6** [16,17,18,19,20,21] and BMS-962212 [15] (Figure 1). Nevertheless, small molecule FXIa inhibitors’ research remained in clinical phase. Further development of novel small molecule FXIa inhibitors and investigation of their Structure-Activity Relationships (SAR) are required. It was reported compound **3** exhibited good FXIa affinity activities (FXIa Ki = 2 nM) but short t_1/2_ lives and other drawbacks.

**Figure 2:**
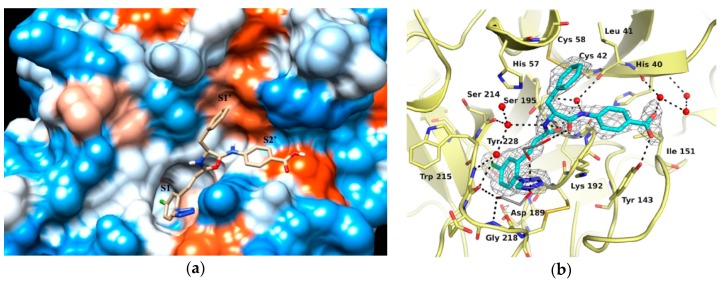
(**a**) Crystal structure of compound **3** in complex with FXIa (Hydrophobicity surface, PDB ID 5E2O); (**b**) Crystal structure of compound **3** in complex with FXIa (Binding mode) [18].

As shown in Figure 2a, the structure **3** binds to FXIa in S1-S1′-S2′ mode. P1, P1 prime (P1′) and P2 prime (P2′) moieties of compound **3** occupy the S1, S1′and S2′ pocket of FXIa (Figure 2a). The carbonyl groups of the cinnamide linker in the P1 and P1′ moieties are well positioned within the oxyanion hole by forming key hydrogen bonds with Ser195 residue (Figure 2b); In addition, the amide NH functional group forms a water mediated hydrogen bond with Leu41 [18]. It’s obvious that the mentioned two linkers are crucial to ligands’ binding mode and it’s necessary to retain the two linkers in the structure optimization.

**Figure 3:**
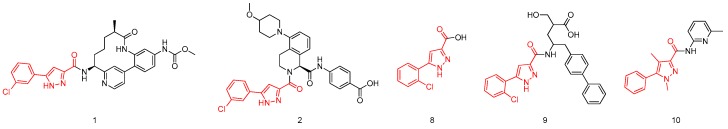
Representative pyrazole derivatives.

It’s summarized that some known FXIa inhibitors such as compound **3**, **4**, **6** (Figure 1) were consisted of 4 parts—P1, P1 prime (P1′), P2 prime (P2′) moieties and scaffold. It’s the molecule which was developed as a FXIa inhibitor and each part of the molecule was necessary but might be not crucial for the overall activity generally. Therefore, it’s reasonable to choose an useful building block as part of FXIa inhibitors. Meanwhile, it is shown clearly that compounds **1**–**2** and **8**–**10** (Figure 3) were all based on the 5-phenyl-1*H*-pyrazole-3-carboxylic acid building block. Of them, compound **8** was a tissue-nonspecific alkaline phosphatase (TNAP) inhibitor [22], compound **9** was an enkephalinase inhibitor [23], compound **10** was a mGlu5 receptor negative allosteric modulator and compounds **1** and **2** both were FXIa inhibitors [16,17,24]. Thus, novel FXIa inhibitors might be developed based on the excellent 5-phenyl-1*H*-pyrazole-3-carboxylic acid derivatives by using the Fragment Based Lead Generation strategy. 

**Figure 4:**
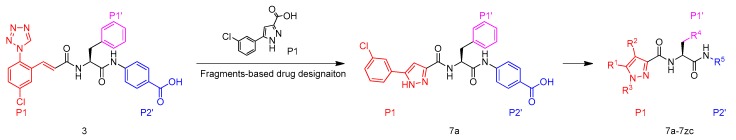
Design of FXIa inhibitors in present study.

Furthermore, it was known that structure optimization using an optimal neutral P1 moiety could significantly facilitate identification of a potent, orally bioavailable FXIa inhibitor [25]. Taking these points into account, we replaced the P1 moiety in compound **3** with more neutral chloro-benzene leading to compound **7a** and further modification of P1, P1′ and P2′ moieties in **7a** furnished a series of 5-phenyl-1*H*-pyrazole-3-carboxamide FXIa inhibitors, of which the SAR was then systematically investigated (Figure 4).

## 2. Results and Discussion

### 2.1. Chemistry

**Scheme 1:**
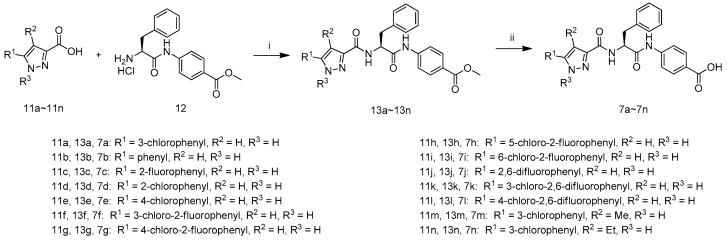
Synthetic route of compounds **7a**–**7n**. *Reagents and conditions*: (i) HOBT, EDCI, DIEA, DMF, r.t.; (ii) (1) LiOH·H_2_O, MeOH, H_2_O, 40 °C; (2) 1 M hydrochloric acid.

The synthetic route to target compounds **7a**–**7n** is shown in Scheme 1. The coupling of compound **12** with a series of carboxylic acids **11a**–**11n** afforded **13a**–**13n** in the presence of 1-hydroxybenzotriazole, *N*-(3-dimethylaminopropyl)-*N*′-ethylcarbodiimide hydrochloride and *N*,*N*-diisopropyl-ethylamine in DMF, which weretreated with LiOH·H_2_O in aqueous methanol at 40 °C and acidified with 1 M hydrochloric acid to pH 3–4 to afford target compounds **7a**–**7n**.

**Scheme 2:**
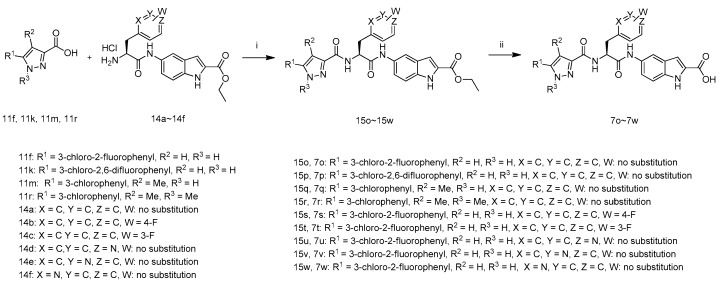
Synthetic route of compounds **7o**–**7w**. *Reagents and conditions*: (i) HOBT, EDCI, DIEA, DMF, r.t.; (ii) (1) LiOH·H_2_O, EtOH, H_2_O, 40 °C; (2) 1 M hydrochloric acid.

The synthetic route to target compounds **7o**–**7w** is summarized in Scheme 2. The intermediates **15o**–**15w** were prepared by the coupling of carboxylic acids **11f**, **11k**, **11m**, **11r** with amines **14a**–**14f** in the presence of 1-hydroxybenzotriazole, *N*-(3-dimethylaminopropyl)-*N*′-ethylcarbodiimide hydrochloride and *N*,*N*-diisopropylethylamine in DMF, which were treated with LiOH·H_2_O in aqueous ethanol at 40 °C. The resultant mixture was acidified with 1 M hydrochloric acid to pH 3–4 to afford target compounds **7o**–**7w**.

**Scheme 3:**
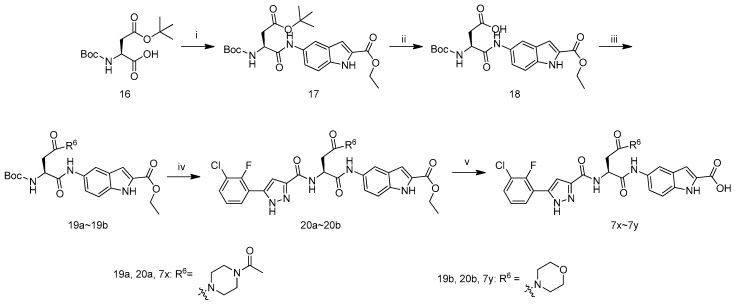
Synthetic route of compounds **7x**–**7y**. *Reagents and conditions*: (i) POCl_3_, Pyridine, ethyl 5-amino-1*H*-indole-2-carboxylate, 0 °C; (ii) (1) TFA: CH_2_Cl_2_ = 1:1; (2) (Boc)_2_O, Et_3_N, CH_2_Cl_2_; (iii) for **19a**: 1-(piperazin-1-yl)ethanone, HOBT, EDCI, DIEA, DMF, r.t.; for **19b**: morpholine, HOBT, EDCI, DIEA, DMF, r.t.; (iv) (1) HCl/EtOAc, r.t.; (2) **11f**, HOBT, EDCI, DIEA, DMF, r.t.; (v) (1) LiOH·H_2_O, EtOH, H_2_O, r.t.; (2) 1 M hydrochloric acid.

The synthetic route to target compounds **7x**–**7y** is depicted in Scheme 3. Boc-Asp(OtBu)-OH (**16**) first reacted with ethyl 5-amino-1*H*-indole-2-carboxylate in the presence of POCl_3_ and pyridine in CH_2_Cl_2_ to give the adduct **17**, which was deprotected with TFA/CH_2_Cl_2_ (1/1 by *v*/*v*), and treated with (Boc)_2_O and Et_3_N in CH_2_Cl_2_ to afford compound **18**. The coupling of carboxylic acid **18** with 1-(piperazin-1-yl)ethanone or morpholine yielded **19a** or **19b** in the presence of1-hydroxybenzotriazole, *N*-(3-dimethylaminopropyl)-*N*′-ethylcarbodiimide hydrochloride and *N*,*N*-diisopropylethylamine in DMF.Removal of *N*-Boc in **19a** with hydrochloric ethyl acetate and the subsequent reaction with **11f** in the presence of1-hydroxybenzotriazole, *N*-(3-dimethyl-aminopropyl)-*N*′-ethylcarbodiimide hydrochloride and *N*,*N*-diisopropylethylamine in DMF yielded **20a**. Hydrolysis of **20a** with LiOH·H_2_O in aqueous ethanol and acidification with 1 M hydrochloric acid afforded **7t**. Compounds **20b** and **7u** were prepared according to the procedure described for the preparation of **20a** and **7t**.

**Scheme 4:**
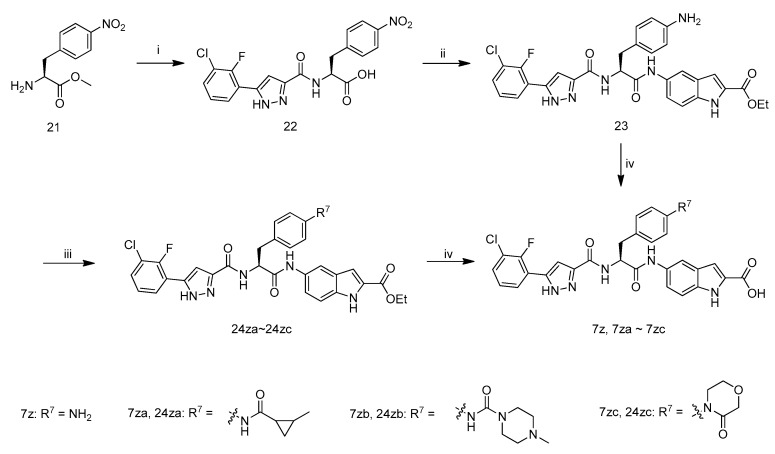
Synthetic route of compounds **7z**–**7zc**. *Reagents and conditions*: (i) (1) 11f, HOBT, EDCI, DIEA, DMF, r.t.; (2) LiOH·H_2_O, MeOH, H_2_O, r.t.; (3) 1 M hydrochloric acid; (ii) (1) ethyl 5-amino-1*H*-indole-2-carboxylate, HOBT, EDCI, DIEA, DMF, r.t.; (2) Pd/C, H_2_, MeOH, EtOAc; (iii) for **24za**: 2-Methylcyclopropanecarboxylic acid, POCl_3_, Pyridine, CH_2_Cl_2_, −10 °C; for **24zb**: 4-methylpiperazine-1-carbonyl chloride, Pyridine, DMAP, DMF; for **24zc**: (1) 2-(2-chloroethoxy)acetic acid, EEDQ, THF; (2) NaH, THF; (iv) (1) LiOH·H_2_O, MeOH, H_2_O, 40 °C; (2) 1 M hydrochloric acid.

The synthetic route to target compounds **7z**, **7za**–**7zc** is illustrated in Scheme 4. Coupling of carboxylic acid **11f** with amine **21** in the presence of 1-hydroxybenzotriazole, *N*-(3-dimethyl-aminopropyl)-*N*′-ethylcarbodiimide hydrochloride and *N*,*N*-diisopropylethylamine in DMF at room temperature, followed by the hydrolysiswithLiOH·H_2_O in aqueous methanol at room temperature, and subsequent acidification with 1 M hydrochloric acid afforded compound **22**. The coupling of compound **22** with ethyl 5-amino-1*H*-indole-2-carboxylate in the presence of POCl_3_ and pyridine in CH_2_Cl_2_, and next reduction of NO_2_ with H_2_ (1 atm) in MeOH and ethyl acetate in the presence of Pd/C yielded compound **23**. Hydrolysis of ethyl ester with LiOH·H_2_O in aqueous ethanol at 40 °C, and acidification of the resultant mixture with 1 M hydrochloric acid afforded target compounds **7z**.Compound **23** reacted with 2-methylcyclopropanecarboxylic acid in the presence of POCl_3_ and pyridine in CH_2_Cl_2_ leading to **24za**. Reaction of **23** with 4-methylpiperazine-1-carbonyl chloride in the presence of pyridine and 4-dimethylaminopyridine in DMF afforded **24zb**. Compound **23** was coupled with 2-(2-chloroethoxy)acetic acid in the presence of *N*-ethoxycarbonyl-2-ethoxy-1,2-dihydroquinoline in THF, and the resultant product was treated with NaH in THF leading to **24zc**. Target compounds **7za**–**7zc** were synthesized from **24za**–**24zc** according to the procedure described for the preparation of **7z**.

**Scheme 5:**
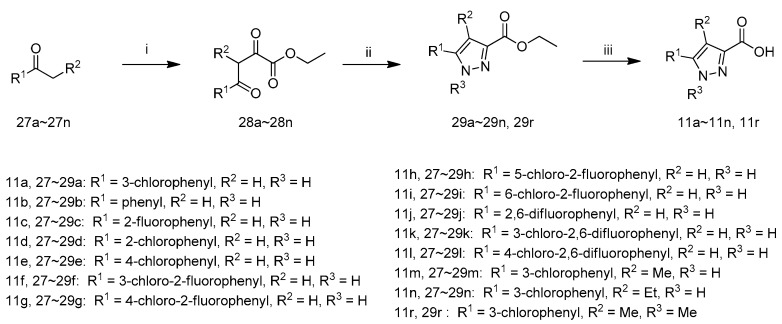
Synthetic route of compounds **11a**~**11n** and **11r**. *Reagents and conditions*: (i) (1) LiHMDS, Diethyl oxalate, MTBE, 0 °C; (2) 1 M hydrochloric acid; (ii) for **29a**–**29n**: hydrazine hydrate, EtOH, 80 °C; for **29r**: Methylhydrazine sulfate, EtOH, 80 °C; (iii) (1) NaOH, EtOH, H_2_O, r.t.; (2) 1 M hydrochloric acid.

The synthetic route to intermediates **11a**–**11n** and **11r** is shown in Scheme 5 according to relevant references [26]. **27a** first reacted with diethyl oxalate in the presence ofLiHMDS in MTBE at 0 °C and the resultant mixture was acidified with hydrochloric acid (1 M) to yield **28a**. The cyclization of **28a** with hydrazine hydrate in EtOH at 80 °C yielded **29a**. The hydrolysis of **29a** with NaOH in EtOH and H_2_O and acidification with 1 M hydrochloric acid afforded **11a**. The intermediates **11b**–**11n** and **11r** were synthesized from **27b**–**27n** according to the procedure described for the preparation of **11a**.

**Scheme 6:**
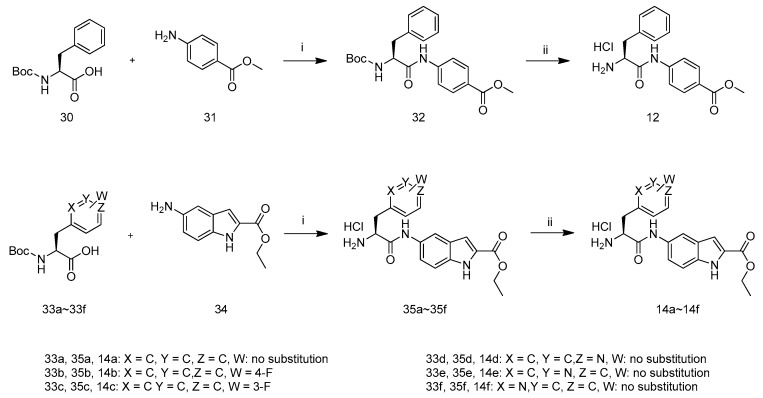
Synthetic route of compounds **14a**–**14f** and **12**. *Reagents and conditions*: (i) POCl_3_, Pyridine, CH_2_Cl_2_, −10 °C; (ii) HCl/EtOAc.

The synthetic route to intermediates **14a**–**14f** and**12** is depicted in Scheme 6 according to relevant references [18]. Compound **32** was prepared by the coupling of carboxylic acid **30** with amine **31** in the presence of POCl_3_ and pyridine in CH_2_Cl_2_ at −10 °C. Removal of *N*-Boc of **32** with hydrochloric acid in ethyl acetate yielded compound **12**. The preparation of compound **14a**–**14f** followed the procedure described for the preparation of compound **12**.

### 2.2. Biological Activity and Discussion

#### 2.2.1. In Vitro Inhibition Activity Studies on FXIa

In vitro FXIa affinity assay was used to examine the potency of **7a**–**7zc** against FXIa. As the effects of compound **4**, compound **3** and BMS-962212 (Figure 1) on FXIa activities were almost in the same degree (FXIa Ki = 0.7~3 nm) and they were all reported by Bristol-Myers Squibb Company, so whichever one was chosen as a positive control for in vitro FXIa affinity assays and activated partial prothrombin time (aPTT) coagulation assays is reasonable. Taking these points into account, compound **4** was used as a positive control in both assays. As shown in Table 1, **7a**, **7f**, **7k**, **7m** showed better potency for FXIa’s inhibition than others in series of **7a**–**7n** with the same P1′, P2′moiety and scaffold. Furthermore, **7m** showed weaker potency for FXIa’s inhibition than **7a** but better potency than **7n**. Therefore, it can be inferred that **7m**’s -Me is a relatively better R_2_ substitution than **7a**’s -H and **7n**’s -Et for FXIa’s inhibition. For further optimization, the P2′ moiety in**7f**, **7k** and **7m** was changed from the 4-aminobenzoic acid moiety to 5-amino-1*H*-indole-2-carboxylic acid moiety to give **7o**, **7p** and **7q**, respectively, of which **7o** possessed the best potency for FXIa’s inhibition. Meanwhile, we also employed **7r**’s inhibition potency of FXIa which is different from **7q** at R_3_ position (-Me substitution), and the results suggested that the R_3_ substitution plays a minor role in the FXIa’s inhibition. Based on above results, **7o** was chosen for further modification and the P1′moiety was replaced by 4 other groups. It’s indicated that replacement of P1′ moiety on **7o**’ benzene with 4-fluorobenzo (**7s**) and 3-fluorobenzo (**7t**) improved the potency slightly (**7s** = 930.49 nm, **7t** = 1.66 μm, **7o** = 1.97 μm). The replacement of benzyl-type P1′ moiety in **7o** with 4-pyridine, 3-pyridine or 2-pyridine (**7u**, **7v**, **7w**, respectively) was not benefit for potency as **7v**, **7u**, and **7w** showed reduced potency than **7o** (**7o** = 1.97 μm, **7v** = 3.52 μm, **7u** = 13.27 μm, **7w** = 8.99 μm). The replacement of the P1′ moiety with carboxamide (**7x**, **7y**) was harmful for potency with 15 times reduction on potency (**7o** = 1.97 μm, **7x** = 81.18 μm, **7y** = 64.01 μm). At last, replacement of the P1′ moiety in **7o** with 2-methylcyclopropanecarboxamide (**7za**) obviously improved the potency for more than 20 times (**7za** = 90.37 nm, **7o** = 1.97 μm). In addition, **7za** shows similar FXIa inhibitory potency as previously reported compound **4**(**7za** = 90.37 nm, **4** = 23.48 nm).

#### 2.2.2. Activated Partial Prothrombin Time (aPTT) In Vitro Coagulation Assays

In order to assess the in vitro coagulation activity of compound **7za**, activated partial prothrombin time (aPTT) of **7za** and compound **4** was compared. As indicated in Table 2, **7za** showed good in vitro coagulation activity with 1.5× aPTT value of 43.33 μM in rabbit plasma.

### 2.3. Molecular Model Studies on the Interaction of Compound ***7za*** with FXIa

**Figure 5:**
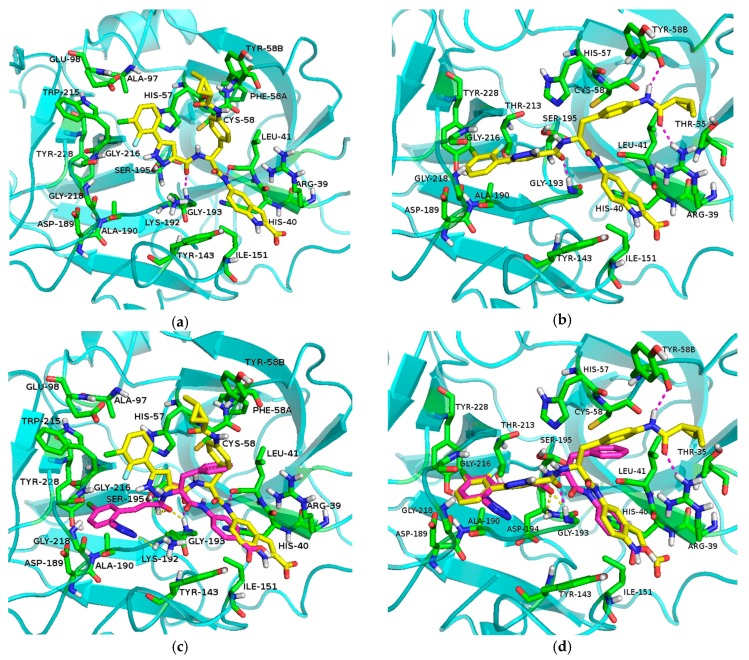
(**a**) Molecular model 1 of **7za** in active site of FXIa; (**b**) Molecular model 2 of **7za** in active site of FXIa; (**c**) Superposition of molecular mode 1 of **7za** with compound **3** (**7za**: yellow, compound **3**: purple); (**d**) Superposition of molecular mode 2 of **7za** with compound **3** (**7za**: yellow, compound **3**: purple).

Molecular docking method was used to study the binding mode of compound **7za** in the active site of FXIa. Two different binding modes of compound **7za** with FXIa were obtained (Figure 5a,b). Superposition of these two binding modes with the known compound **3** (Figure 5c,d) showed that **7za** in the second binding mode and known compound **3** overlay well (Figure 5d), indicating that this kind of binding model of **7za** with FXIa is more reasonable (Figure 5b). As designated, the 5-(3-chloro-2-fluorophenyl)-1*H*-pyrazole moiety is located deeply inS1 pocket of FXIa with the Cl atom forming an interaction with Tyr228, and the carbonyl group of 5-(3-chloro-2-fluorophenyl)-1*H*-pyrazole-3-carboxamide is well positioned within the oxyanion hole by forming key hydrogen bonds with Gly193 residues which is similar to the binding model of compound **3** with FXIa (Figure 5d). Furthermore, the 2-methylcyclopropanecarboxamide group of **7za** makes 2 direct hydrogen bonds with Tyr58B and Thr35 in the FXIa backbone, making **7za** binds to FXIa in a highly efficient manner (Figure 5b).

## 3. Materials and Methods

### 3.1. General Information

Reagents and solvents were purchased from commercial suppliers and used without further purification. Reactions were monitored by thin layer chromatography. ^1^H-NMR spectra (400 MHz) and ^13^C-NMR (100 MHz) were recorded for DMSO-d_6_ solutions on a Bruker spectrometer (Bruker, Billerica, MA, USA). MS were measured on a Finnigan LCQ Mass (Thermo Fisher Scientific, Waltham, MA, USA). HRMS were measured on a TOF LC/MS instrument (Agilent Technologies, Santa Clara, CA, USA).

### 3.2. Chemistry

*Ethyl 2,4-dioxo-4-phenylbutanoate* (**28b**): To a stirred solution of acetophenone (**27b**) (2.0 g, 16.5 mmol) in MTBE (30 mL) was added lithium hexamethyldisilazide (1.3 M, 12.7 mL, 16.5 mmol) dropwise at 0 °C; After addition, the reaction mixture was stirred at 0 °C for 0.5 h and diethyl oxalate (3.0 g, 20.8 mmol) was added dropwise. Then, the mixture was stirred at room temperature overnight. TLC analysis showed reaction was complete and the reaction mixture was extracted with H_2_O (20 mL). The aqueous layer was separated, acidified by hydrochloric acid (1 M) to pH 6 and extracted by ethyl acetate (10 mL × 2). The combined organic layer was concentrated in vacuum to give **28b** as yellow oil, which was used for next step without further purification (3.4 g, 92.7% yield).

*Ethyl3-phenyl-1H-pyrazole-5-carboxylate* (**29b**): To a solution of **28b** (3.4 g, 15.4 mmol) in EtOH (15 mL) was added hydrazine hydrate (1.2 g, 24.0 mmol) and the mixture was stirred at 50 °C for 2 h when TLC analysis indicated completion of reaction. Then the reaction mixture was evaporated to get crude **29b** as brown oil, which was used for next step without further purification (2.6 g, 77.9% yield).

*5-Phenyl-1H-pyrazole-3-carboxylic acid* (**11b**): To a solution of compound **29b** (2.6 g, 9.1 mmol) in MeOH (30 mL) and H_2_O (15 mL) was added LiOH·H_2_O (0.5 g, 20.0 mmol) and the mixture was stirred at 70 °C for 8 h. The reaction mixture was evaporated and H_2_O (15 mL) was added, then acidified by hydrochloric acid (1 M) to pH 3. The suspension was filtered and washed by H_2_O (10 mL), dried at 50 °C for 4 h to afford **11b** as a white solid (2.1 g, 89.5% yield), m.p.: 227–229 °C, decomposition. ^1^H-NMR: δ 13.39 (s, 1H), 7.83 (m, 2H), 7.41–7.45 (m, 2H), 7.31–7.35 (m, 1H), 7.18(s, 1H). HRMS (ESI) calcd. For C_10_H_8_ClN_2_O_2_^+^: [M + H]^+^ *m*/*z*: 189.0659, found: 189.0659. The ^1^H-NMR data were in good agreement with those reported [26].

Compounds **11a**, **11r** and **11c**–**11n** were synthesized according to the procedure described for the preparation of **11b**.

*5-(3-Chlorophenyl)-1H-pyrazole-3-carboxylic acid* (**11a**): white solid product (1.5 g, 47.9% yield), m.p.: 219–221 °C, decomposition. ^1^H-NMR: δ 13.85–13.15 (m, 2H), 7.90 (m, 1H), 7.82–7.80 (d, *J* = 7.6 Hz, 1H), 7.47–7.37 (m, 2H), 7.29 (s, 1H). HRMS (ESI) calcd. For C_10_H_9_N_2_O_2_^+^: [M + H]^+^ *m*/*z*: 223.0269, found: 223.0268. The ^1^H-NMR data were in good agreement with those reported [26].

*5-(3-Chlorophenyl)-1H-pyrazole-3-carboxylic acid* (**11a**): white solid product (1.5 g, 47.9% yield), m.p.: 219–221 °C, decomposition. ^1^H-NMR: δ 13.85–13.15 (m, 2H), 7.90 (m, 1H), 7.82–7.80 (d, *J* = 7.6 Hz, 1H), 7.47–7.37 (m, 2H), 7.29 (s, 1H). HRMS (ESI) calcd. For C_10_H_9_N_2_O_2_^+^: [M + H]^+^ *m*/*z*: 223.0269, found: 223.0268. The ^1^H-NMR data were in good agreement with those reported [26].

*3-(2-Fluorophenyl)-1H-pyrazole-5-carboxylic acid* (**11c**): white solid product (2.3 g, 77.0% yield), m.p.: 232–234 °C, decomposition. ^1^H-NMR: δ 13.84–13.52 (m, 2H), 7.94–7.91 (m, 1H), 7.43–7.38 (m, 1H), 7.34–7.27 (m, 2H), 7.05–7.04 (d, *J* = 3.6 Hz, 1H). HRMS (ESI) calcd. For C_10_H_8_FN_2_O_2_^+^: [M + H]^+^ *m*/*z*: 207.0564, found: 207.0563 [27].

*3-(2-Chlorophenyl)-1H-pyrazole-5-carboxylic acid* (**11d**): white solid product (1.8g, 62.5% yield), m.p.: 229–231 °C, decomposition. ^1^H-NMR: δ 13.64–13.49 (m, 2H), 7.74 (s, 1H), 7.57–7.55 (m, 1H), 7.44–7.41 (m, 2H), 7.12 (s, 1H). HRMS (ESI) calcd. For C_10_H_8_ClN_2_O_2_^+^: [M + H]^+^ *m*/*z*: 223.0269, found: 223.0268 [27].

*3-(4-Chlorophenyl)-1H-pyrazole-5-carboxylic acid* (**11e**): white solid product (1.5 g, 52.1% yield), m.p.: 240–242 °C, decomposition. ^1^H-NMR: δ 13.86–13.64 (m, 2H), 7.86–7.84 (d, *J* = 8.4 Hz, 2H), 7.49–7.47 (d, *J* = 8.4 Hz, 2H), 7.04 (s, 1H). HRMS (ESI) calcd. For C_10_H_8_ClN_2_O_2_^+^: [M + H]^+^ *m*/*z*: 223.0269, found: 223.0269. The ^1^H-NMR data were in good agreement with those reported [26].

*3-(3-Chloro-2-fluorophenyl)-1H-pyrazole-5-carboxylic acid* (**11f**): white solid product (2.0 g, 71.7% yield), m.p.: 236–238 °C, decomposition. ^1^H-NMR: δ 13.77 (m, 1H), 7.92–7.88 (m, 1H), 7.56–7.52 (m, 1H), 7.30–7.26 (m, 1H), 6.98–6.97 (d, *J* = 4.0 Hz, 1H). HRMS (ESI) calcd. For C_10_H_7_ClFN_2_O_2_^+^: [M + H]^+^ *m*/*z*: 241.0175, found: 241.0173.

*3-(4-Chloro-2-fluorophenyl)-1H-pyrazole-5-carboxylic acid* (**11g**): white solid product (1.9 g, 68.1% yield), m.p.: 234–236 °C, decomposition. ^1^H-NMR: δ 13.07 (s, 1H), 7.98–7.93 (t, *J* = 8.4 Hz, 2H), 7.47–7.44 (qd, *J* = 2 Hz, 1H), 7.31–7.28 (qd, *J* = 2 Hz, 1H), 6.66–6.65 (d, *J* = 4.4 Hz, 1H). HRMS (ESI) calcd. For C_10_H_7_ClFN_2_O_2_^+^: [M + H]^+^ *m*/*z*: 241.0175, found: 241.0172.

*3-(5-Chloro-2-fluorophenyl)-1H-pyrazole-5-carboxylic acid* (**11h**): white solid product (1.5 g, 53.8% yield), m.p.: 229–231 °C, decomposition. ^1^H-NMR: δ 14.11–13.42 (m, 2H), 7.97–7.95 (m, 1H), 7.47–7.36 (m, 2H), 7.10–7.05 (m, 1H). HRMS (ESI) calcd. For C_10_H_7_ClFN_2_O_2_^+^: [M + H]^+^ *m*/*z*: 241.0175, found: 241.0172.

*3-(2-Chloro-6-fluorophenyl)-1H-pyrazole-5-carboxylic acid* (**11i**): white solid product (1.6 g, 57.4% yield), m.p.: 182–184 °C, decomposition. ^1^H-NMR: δ 14.13–14.06 (m, 1H), 13.72–13.48 (m, 1H), 7.49–7.46 (m, 2H), 7.35 (m, 1H), 6.91 (s, 1H). HRMS (ESI) calcd. For C_10_H_7_ClFN_2_O_2_^+^: [M + H]^+^ *m*/*z*: 241.0175, found: 241.0170 [28].

*3-(2,6-Difluorophenyl)-1H-pyrazole-5-carboxylic acid* (**11j**): white solid product (1.6 g, 55.7% yield), m.p.: 232–334 °C, decomposition. ^1^H-NMR: δ 14.08–13.05 (m, 2H), 7.53–7.46 (m, 1H), 7.25–7.21 (m, 2H), 6.98 (s, 1H). HRMS (ESI) calcd. For C_10_H_7_F_2_N_2_O_2_^+^: [M + H]^+^ *m*/*z*: 225.0470, found: 241.0465.

*3-(3-Chloro-2,6-difluorophenyl)-1H-pyrazole-5-carboxylic acid* (**11k**): white solid product (1.2 g, 44.2% yield), m.p.: 233–235 °C, decomposition. ^1^H-NMR: δ 14.17 (s, 1H), 7.72–7.67 (m, 1H), 7.33–7.29 (m, 1H), 7.02 (s, 1H). HRMS (ESI) calcd. For C_10_H_6_ClF_2_N_2_O_2_^+^: [M + H]^+^ *m*/*z*: 259.0080, found: 259.0074.

*3-(4-Chloro-2,6-difluorophenyl)-1H-pyrazole-5-carboxylic acid* (**11l**): white solid product (1.6 g, 58.9% yield), m.p.: 238–240 °C, decomposition. ^1^H-NMR: δ 13.64 (s, 1H), 7.51–7.49 (d, *J* = 8.0 Hz, 2H), 6.99 (s, 1H). HRMS (ESI) calcd. For C_10_H_6_ClF_2_N_2_O_2_^+^: [M + H]^+^ *m*/*z*: 259.0080, found: 259.0078.

*5-(3-Chlorophenyl)-4-methyl-1H-pyrazole-3-carboxylic acid* (**11m**): white solid product (1.1 g, 39.2% yield), m.p.: 226–228 °C, decomposition. ^1^H-NMR: δ 13.64–13.57 (m, 2H), 7.66 (s, 1H), 7.63–7.57 (m, 1H), 7.51–7.42 (m, 2H), 2.39(s, 3H). HRMS (ESI) calcd. For C_11_H_10_ClN_2_O_2_^+^: [M + H]^+^ *m*/*z*: 237.0425, found: 237.0424.

*5-(3-Chlorophenyl)-4-ethyl-1H-pyrazole-3-carboxylic acid* (**11n**): white solid product (0.2 g, 7.3% yield), m.p.: 236–238 °C, decomposition. ^1^H-NMR: δ 13.43 (s, 1H), 7.58 (s, 1H), 7.53–7.43 (m, 3H), 2.82–2.76 (qd, *J* = 7.2 Hz, 2H), 1.12–1.08 (m, 3H). HRMS (ESI) calcd. For C_12_H_12_ClN_2_O_2_^+^: [M + H]^+^ *m*/*z*: 251.0582, found: 251.0577.

*5-(3-Chlorophenyl)-1,4-dimethyl-1H-pyrazole-3-carboxylic acid* (**11r**): white solid product (1.1 g, 37.0% yield), m.p.: 147–149 °C, decomposition. ^1^H-NMR: δ 12.52 (s, 1H), 7.56–7.54 (m, 3H), 7.41–7.39 (m, 1H), 3.74 (s, 3H), 2.10 (s, 3H). The structure was confirmed by NOESY.HRMS (ESI) calcd. For C_12_H_12_ClN_2_O_2_^+^: [M + H]^+^ *m*/*z*: 251.0582, found: 251.0575.

*(S)-Methyl-4-(2-((tert-butoxycarbonyl)amino)-3-phenylpropanamido)benzoate* (**32**): To a stirred mixture of Boc-Phe-OH (30) (10.0 g, 37.7mmol), methyl 4-aminobenzoate (**31**) (5.7 g, 37.7 mmol) and pyridine (10 mL) in CH_2_Cl_2_ (100 mL) at −10 °C was added POCl_3_ (5.8 g, 37.7 mmol) dropwise.After addition, the reaction mixture was stirred at 0 °C for 2 h when TLC analysis indicated completion of reaction, then H_2_O (20 mL) was added and the organic layer was separated, washed by hydrochloric acid (1 M, 20 mL), dried by Na_2_SO_4_, filtered. The filtrate was evaporated in vacuum to get crude **32** as a yellow solid, which was used for next step without further purification (11.6 g, 77.3% yield).

*(S)-Methyl-4-(2-amino-3-phenylpropanamido)benzoate hydrochloride* (**12**): To a solution of compound **32** (11.5 g, 28.9 mmol) in ethyl acetate (50 mL) was added hydrochloric/ethyl acetate (50 mL, saturated solution), and the mixture was stirred at room temperature overnight. Then TLC analysis showed reaction was complete. The suspension was filtered and the filter cake was washed by ethyl acetate (20 mL), dried at 50 °C for 4 h to afford **12** as a pink solid (8.2 g, 84.3% yield), m.p.: 127–129 °C. ^1^H-NMR: δ 11.27 (s, 1H), 8.45 (s, 3H), 7.93–7.91 (d, *J* = 8.4 Hz, 2H), 7.73–7.71 (d, *J* = 8.8 Hz, 2H), 7.32–7.23 (m, 5H), 4.34 (m, 1H), 3.81 (s, 3H), 3.24–3.12 (m, 2H). ESI-MS (*m*/*z*) = 299.09 [M +H]^+^.

*(S)-Ethyl 5-(2-((tert-butoxycarbonyl)amino)-3-phenylpropanamido)-1H-indole-2-carboxylate* (**35a**): To a mixture of Boc-Phe-OH (**33a**) (5 g, 18.9 mmol), ethyl 5-amino-1*H*-indole-2-carboxylate (**34**) (3.9 g, 18.9 mmol) and pyridine (5 mL) in CH_2_Cl_2_ (50 mL) at −10 °C was added POCl_3_ (2.9 g, 18.9 mmol) dropwise. After addition, the reaction mixture was stirred at −10 °C for 2 h when TLC analysis indicated completion of reaction, then H_2_O (10 mL) was added and the organic layer was separated, washed by hydrochloric acid (1 M, 10 mL), dried by Na_2_SO_4_, filtered. The filtrate was evaporated in vacuum to get crude **35a** as a brown solid, which was used for next step without further purification (6.6 g, 77.6% yield).

*(S)-Ethyl 5-(2-amino-3-phenylpropanamido)-1H-indole-2-carboxylate hydrochloride* (**14a**): To a solution of compound **35a** (6.5 g, 14.4 mmol) in ethyl acetate (15 mL) was added hydrochloric/ethyl acetate (30 mL, saturated solution), and the mixture was stirred at room temperature overnight. Then TLC analysis showed reaction was complete. The suspension was filtered and the filter cake was washed by ethyl acetate (20 mL) and dried at 50 °C for 4 h to afford **14a** as a grey solid (4.9 g, 87.7% yield), m.p.: 135–137 °C, decomposition. ^1^H-NMR: δ 11.86 (s, 1H), 10.46 (s, 1H), 8.36–8.32 (m, 4H), 7.91 (s, 1H), 7.33–7.12 (m, 8H), 4.35–4.29 (m, 2H), 4.19 (m, 1H), 3.13–3.08 (m, 2H), 1.34–1.31 (m, 3H). ESI-MS (*m*/*z*) = 352.10 [M + 1]^+^ [18].

Compounds **14b**~**14f** were synthesized according to the procedure described for the preparation of **14a**.

*(S)-Ethyl5-(2-amino-3-(4-fluorophenyl)propanamido)-1H-indole-2-carboxylatehydrochloride* (**14b**): grey solid product (4.0 g, 52.3% yield), m.p.: 243–245 °C, decomposition. ^1^H-NMR: δ 11.85 (s, 1H), 10.84 (s, 1H), 8.45 (s, 2H), 7.96 (s, 1H), 7.41–7.36 (m, 4H), 7.15–7.11 (m, 3H), 4.36–4.29 (m, 2H), 3.15–3.09 (m, 2H), 1.34–1.30 (m, 3H). ESI-MS (*m*/*z*) = 370.06 [M + 1]^+^ [29].

*(S)-Ethyl 5-(2-amino-3-(3-fluorophenyl)propanamido)-1H-indole-2-carboxylatehydrochloride* (**14c**): grey solid product (3.7 g, 48.6% yield), m.p.: 154–156 °C, decomposition. ^1^H-NMR: δ 11.86 (s, 1H), 10.77 (s, 1H), 8.43 (s, 3H), 7.95 (s, 1H), 7.41–7.32 (m, 3H), 7.22–7.05 (m, 4H), 4.35–4.02 (m, 3H), 3.37–3.11 (m, 2H), 1.35–1.31 (t, *J* = 6.8 Hz, 3H). ESI-MS (*m*/*z*) = 370.02 [M + 1]^+^, HRMS (ESI) calcd. For C_20_H_2__1_FN_3_O_3_^+^: [M + H]^+^ *m*/*z*: 370.1561, found: 370.1553.

*(S)-Ethyl 5-(2-amino-3-(pyridin-4-yl)propanamido)-1H-indole-2-carboxylatehydrochloride* (**14d**): grey solid product (0.5 g, 6.8%), m.p.: 171–173 °C, decomposition. ^1^H-NMR: δ 11.87 (s, 1H), 11.32 (s, 1H), 8.88–8.86 (d, *J* = 6.4 Hz, 2H), 8.59 (s, 3H), 8.13–8.11 (d, *J* = 5.6 Hz, 2H), 8.03 (s, 1H), 7.47–7.40 (m, 2H), 7.12 (s, 1H), 4.57 (s, 1H), 4.35–4.29 (qd, *J* = 6.8 Hz and 7.2 Hz, 2H), 3.65–3.62 (m, 1H), 3.41–3.35 (m, 1H), 1.34–1.30 (t, *J* = 7.2 Hz, 3H). ESI-MS (*m*/*z*) = 353.00 [M + 1]^+^, HRMS (ESI) calcd. For C_19_H_2__1_N_4_O_3_^+^: [M + H]^+^ *m*/*z*: 353.1608, found: 353.1602.

*(S)-Ethyl 5-(2-amino-3-(pyridin-3-yl)propanamido)-1H-indole-2-carboxylatehydrochloride* (**14e**): grey solid product (1.5 g, 19.2%). ^1^H-NMR: δ 11.90 (s, 1H), 11.07 (s, 1H), 8.89 (s, 1H), 8.79–8.77 (d, *J* = 5.6 Hz, 1H), 8.48–8.43 (m, 4H), 8.00 (s, 1H), 7.91–7.88 (t, *J* = 6.4 Hz, 1H), 7.40 (s, 1H), 7.13 (s, 1H), 4.47 (s, 1H), 4.35–4.29 (m, 2H), 3.52–3.47 (m, 1H), 3.32–3.26 (m, 1H), 1.34–1.30 (m, 3H). ESI-MS (*m*/*z*) = 353.15 [M + 1]^+^.

*(S)-Ethyl 5-(2-amino-3-(pyridin-2-yl)propanamido)-1H-indole-2-carboxylatehydrochloride* (**14f**): grey solid product (1.8 g, 24.6%), m.p.: 75–77 °C, decomposition. ^1^H-NMR: δ 11.86 (s, 1H), 10.89 (s, 1H), 8.76–8.75 (d, *J* = 5.2 Hz, 1H), 8.65 (s, 3H), 8.21–8.19 (m, 1H), 7.97 (s, 1H), 7.81–7.79 (d, *J* = 7.6 Hz, 1H), 7.69–7.67 (m, 1H), 7.42–7.38 (m, 2H), 7.11 (s, 1H), 4.60 (s, 1H), 4.35–4.29 (m, 2H), 3.63–3.54 (m, 2H), 1.34–1.30 (t, *J* = 7.2 Hz, 3H). ESI-MS (*m*/*z*) = 353.05 [M + 1]^+^, HRMS (ESI) calcd. For C_19_H_2__1_N_4_O_3_^+^: [M + H]^+^ *m*/*z*: 353.1608, found: 353.1604.

*(S)-Methyl 4-(2-(5-(3-chlorophenyl)-1H-pyrazole-3-carboxamido)-3-phenylpropanamido)benzoate* (**13a**): To a mixture of 3-(3-chlorophenyl)-1*H*-pyrazole-5-carboxylic acid (**14a**, 133 mg, 0.60 mmol) inDMF(4 mL) was added (*S*)-methyl-4-(2-amino-3-phenylpropanamido)benzoate hydrochloride (**12**, 200 mg, 0.60 mmol), *N*,*N*-diisopropyl-ethylamine (232 mg, 1.80 mmol), 1-hydroxybenzotriazole(161 mg, 1.20 mmol) and *N*-(3-dimethylaminopropyl)-*N*′-ethylcarbodiimide hydrochloride (229 mg, 1.20 mmol) and the reaction mixture was stirred at room temperature overnight. Then TLC analysis indicated reaction was complete, and H_2_O (40 mL) was added. The mixture was stirred for 10 min and filtered to get crude product **13a** as a yellow solid, which was used for next step without further purification.

*(S)-4-(2-(5-(3-Chlorophenyl)-1H-pyrazole-3-carboxamido)-3-phenylpropanamido)benzoic acid* (**7a**): To a mixture of compound **13a** (289 mg, 0.60 mmol) in MeOH (3 mL) and H_2_O (1.5 mL) was added LiOH·H_2_O (100 mg, 2.40 mmol) and the reaction mixture was stirred at 40 °C for 6 h when TLC analysis indicated completion of reaction. The mixture was evaporated in vacuum and H_2_O (2 mL) was added, extracted by MTBE (2 mL) and acidified by hydrochloric acid (1 M) to pH 3–4. After stirred for 0.5 h, the suspension was filtered, and the filter cake washed by H_2_O (5 mL) and dried at 50 °C for 2 h to afford **7a** as a white solid (185 mg, 63.4% yield for 2 steps); m.p.: 147–149 °C, decomposition.^1^H-NMR: δ 13.68 (s, 1H), 12.65 (s, 1H), 10.57 (s, 1H), 7.93–7.88 (m, 3H), 7.73–7.71 (m, 2H), 7.60–7.15 (m, 8H),4.93–4.88 (m, 1H), 3.31–3.15 (m, 2H).^13^C-NMR: δ 170.64, 167.03, 142.99, 137.61, 133.84, 130.88, 130.43, 129.34, 129.20, 128.20, 127.94, 126.51, 125.55, 124.88, 123.91, 118.78, 103.51, 55.29, 37.46. ESI-MS (*m*/*z*) = 488.90 [M + H]^+^, HRMS (ESI) calcd. For C_26_H_22_ClN_4_O_4_^+^: [M + H]^+^ *m*/*z*: 489.1324, found: 489.1317.

Compounds **7b**~**7n** were synthesized according to the procedure described for the preparation of **7a**.

*(S)-4-(3-Phenyl-2-(5-phenyl-1H-pyrazole-3-carboxamido)propanamido)benzoic acid* (**7b**): white solid product (183 mg, 67.4% yield), m.p.: 137–139 °C, decomposition.^1^H-NMR: δ 13.68–13.64 (m, 1H), 12.76–12.70 (m, 1H), 10.52 (s, 1H), 8.11–8.09 (m, 1H), 7.91–7.88 (d, *J* = 8.4 Hz, 2H), 7.77–7.70 (m, 4H), 7.46–7.43 (m, 2H), 7.34–7.19 (m, 5H), 7.17–7.09 (m, 1H), 7.06 (s, 1H), 4.93–4.87 (m, 1H), 3.16–3.15 (m, 2H). ^13^C-NMR: δ 171.25, 167.67, 161.28, 143.49, 138.15, 131.10, 129.92, 129.82, 129.62, 128.84, 127.16, 126.24, 125.92, 119.42, 103.42, 55.67, 38.12. ESI-MS (*m*/*z*) = 454.99 [M + H]^+^, HRMS (ESI) calcd. For C_26_H_23_N_4_O_4_^+^: [M + H]^+^ *m*/*z*: 455.1714, found: 455.1703.

*(S)-4-(2-(5-(2-Fluorophenyl)-1H-pyrazole-3-carboxamido)-3-phenylpropanamido)benzoic acid* (**7c**): white solid product (220 mg, 77.9% yield), m.p.: 86–88 °C, decomposition. ^1^H-NMR: δ 13.76–13.69 (m, 1H), 12.79–12.68 (m, 1H), 10.57 (s, 1H), 9.02–8.97 (m, 1H), 7.90–7.88 (d, *J* = 8.8 Hz, 2H), 7.73–7.71 (d, *J* = 8.4 Hz, 2H), 7.39–7.17 (m, 8H), 7.15–7.07 (m, 1H), 7.00 (s, 1H), 4.93–4.87 (m, 1H), 3.23–3.15 (m, 2H). ESI-MS (*m*/*z*) = 472.99 [M + H]^+^. ^13^C-NMR: δ 171.38, 167.65, 160.65, 143.73, 138.34, 130.98, 130.70, 130.01, 129.79, 128.75, 127.08, 126.09, 125.49, 119.39, 116.81, 106.49, 56.03, 37.90. HRMS (ESI) calcd. For C_26_H_22_FN_4_O_4_^+^: [M + H]^+^ *m*/*z*: 473.1620, found: 473.1600.

*(S)-4-(2-(5-(2-Chlorophenyl)-1H-pyrazole-3-carboxamido)-3-phenylpropanamido)benzoic acid* (**7d**): white solid product (221 mg, 75.7% yield), m.p.: 92–94 °C, decomposition. ^1^H-NMR: δ 13.31 (s, 1H), 10.95 (s, 1H), 8.68 (s, 1H), 7.94–7.92 (d, *J* = 8.8 Hz, 2H), 7.84–7.82 (d, *J* = 8.4 Hz, 2H), 7.74–7.72 (d, *J* = 6.8 Hz, 1H), 7.55–7.53 (d, *J* = 7.2 Hz, 1H), 7.42–7.35 (m, 4H), 7.31–7.22 (m, 3H), 7.17–7.14 (m, 1H), 5.01–4.95 (m, 1H), 3.28–3.11 (m, 2H). ^13^C-NMR: δ 172.94, 170.79, 167.03, 143.12, 137.74, 131.15, 130.57, 129.94, 129.40, 129.19, 128.30, 127.53, 126.54, 125.47, 118.82, 106.57, 55.39, 36.41. ESI-MS (*m*/*z*) = 489.01 [M + H]^+^, HRMS (ESI) calcd. For C_26_H_22_ClN_4_O_4_^+^: [M + H]^+^ *m*/*z*: 489.1324, found: 489.1309.

*(S)-4-(2-(5-(4-Chlorophenyl)-1H-pyrazole-3-carboxamido)-3-phenylpropanamido)benzoic acid* (**7e**): white solid product (177 mg, 60.3% yield), m.p.: 174–176 °C, decomposition. ^1^H-NMR: δ 13.74–13.63 (m, 1H), 12.66 (s, 1H), 10.49 (s, 1H), 10.37 (s, 1H), 7.91–7.86 (t, *J* = 8.8 Hz, 2H), 7.80–7.78 (d, *J* = 8.0 Hz, 1H), 7.72–7.67 (m, 2H), 7.51 (s, 2H), 7.27–7.15 (m, 6H), 7.08 (s, 1H), 4.90–4.89 (m, 1H), 3.17–3.15 (m, 2H). ^13^C-NMR: δ 170.59, 168.12, 160.16, 142.37, 137.66, 132.74, 130.36, 129.35, 129.02, 128.23, 127.97, 127.58, 127.04, 126.54, 118.72, 103.22, 55.37, 37.50. ESI-MS (*m*/*z*) = 488.89 [M + H]^+^, HRMS (ESI) calcd. For C_26_H_22_ClN_4_O_4_^+^: [M + H]^+^ *m*/*z*: 489.1324, found: 489.1311.

*(S)-4-(2-(5-(3-Chloro-2-fluorophenyl)-1H-pyrazole-3-carboxamido)-3-phenylpropanamido)benzoic acid* (**7f**): white solid product (258 mg, 84.8% yield), m.p.: 185–187 °C, decomposition. ^1^H-NMR: δ 13.86 (s, 1H,), 12.69 (s, 1H), 10.54 (s, 1H), 10.38 (s, 1H), 8.31–8.29 (d, *J* = 8.0 Hz, 1H), 7.91–7.86 (m, 2H), 7.73–7.67 (m, 2H), 7.57 (s, 1H), 7.35–7.19 (m, 5H), 7.17–7.15 (m, 1H), 4.91–4.89 (m, 1H), 3.16–2.99 (m, 1H), 2.86–2.81 (m, 1H). ^13^C-NMR: δ 170.70, 168.35, 159.87, 155.44, 142.28, 137.77, 130.47, 129.42, 128.36, 128.00, 127.03, 126.68, 125.87, 121.01, 118.85, 106.21, 55.55, 37.46. ESI-MS (*m*/*z*) = 506.93 [M + H]^+^, HRMS (ESI) calcd. For C_26_H_21_ClFN_4_O_4_^+^: [M + H]^+^ *m*/*z*: 507.1230, found: 507.1220.

*(S)-4-(2-(5-(4-Chloro-2-fluorophenyl)-1H-pyrazole-3-carboxamido)-3-phenylpropanamido)benzoic acid* (**7g**): white solid product (220 mg, 72.6% yield), m.p.: 143–145 °C, decomposition. ^1^H-NMR: δ 13.82 (s, 1H), 12.70 (s, 1H), 10.55 (s, 1H), 9.00 (s, 1H), 7.94 (s, 1H), 7.90–7.88 (d, *J* = 8.4 Hz, 2H), 7.73–7.71 (d, *J* = 8.4 Hz, 2H), 7.58–7.56 (m, 1H), 7.35–7.27 (m, 3H), 7.26–7.24 (m, 2H), 7.18–7.15 (m, 1H), 4.93–4.87 (m, 1H), 3.18–3.11 (m, 2H). ^13^C-NMR: δ 171.27, 167.62, 160.54, 158.03, 143.55, 138.26, 133.94, 131.04, 129.91, 128.78, 127.11, 126.15, 125.80, 119.39, 117.65, 106.48, 55.92, 37.90. HRMS (ESI) calcd. For C_26_H_21_ClFN_4_O_4_^+^: [M + H]^+^ *m*/*z*: 507.1230, found: 507.1215.

*(S)-4-(2-(5-(5-Chloro-2-fluorophenyl)-1H-pyrazole-3-carboxamido)-3-phenylpropanamido)benzoic acid* (**7h**): grey solid product (176 mg, 57.9% yield), m.p.: 179–181 °C, decomposition. ^1^H-NMR: δ 13.89–13.83 (m, 1H), 12.78 (s, 1H), 10.55 (s, 1H), 8.78–8.77 (m, 1H), 7.96–7.94 (m, 1H), 7.90–7.88 (d, *J* = 8.8 Hz, 2H), 7.71–7.69 (d, *J* = 8.4 Hz, 2H), 7.47–7.18 (m, 6H), 7.16–7.15 (m, 1H), 4.92–4.87 (m, 1H), 3.19–3.07 (m, 2H). ^13^C-NMR): δ 171.21, 168.05, 159.40, 143.20, 138.31, 130.98, 129.88, 129.5, 128.80, 127.73, 127.11, 119.32, 119.13, 118.89, 106.79, 55.95, 37.90. ESI-MS (*m*/*z*) = 506.87 [M + H]^+^, HRMS (ESI) calcd. For C_26_H_21_ClFN_4_O_4_^+^: [M + H]^+^ *m*/*z*: 507.1230, found: 507.1221.

*(S)-4-(2-(5-(2-Chloro-6-fluorophenyl)-1H-pyrazole-3-carboxamido)-3-phenylpropanamido)benzoic acid* (**7i**): white solid product (189 mg, 62.1% yield), m.p.: 203–205 °C, decomposition. ^1^H-NMR: δ 13.74–13.70 (m, 1H), 10.64 (s, 1H), 7.89–7.87 (d, *J* = 8.8 Hz, 2H), 7.71–7.69 (d, *J* = 8.4 Hz, 2H), 7.54–7.42 (m, 2H), 7.38–7.36 (m, 3H), 7.28–7.24 (m, 2H), 7.19–7.15 (m, 1H), 7.08–6.98 (m, 1H), 4.92–4.86 (m, 1H), 3.21–3.09 (m, 2H). ^13^C-NMR: δ 171.31, 168.49, 162.00, 143.11, 138.33, 134.48, 130.98, 129.94, 128.82, 127.69, 127.14, 126.53, 119.26, 115.63, 115.40, 108.35, 56.03, 37.88. ESI-MS (*m*/*z*) = 506.88 [M + H]^+^, HRMS (ESI) calcd. For C_26_H_21_ClFN_4_O_4_^+^: [M + H]^+^ *m*/*z*: 507.1230, found: 507.1213.

*(S)-4-(2-(5-(2,6-Difluorophenyl)-1H-pyrazole-3-carboxamido)-3-phenylpropanamido)benzoic acid* (**7j**): white solid product (153 mg, 50.6% yield), m.p.: 205–207 °C, decomposition. ^1^H-NMR: δ 13.66 (s, 1H), 12.79–12.70 (m, 1H), 10.54 (s, 1H), 7.91–7.89 (d, *J* = 8.4 Hz, 2H), 7.73–7.70 (d, *J* = 8.4 Hz, 2H), 7.50 (s, 1H), 7.35–7.03 (m, 6H), 7.03–6.99 (m, 2H), 4.91–4.89 (m, 1H), 3.16–3.15 (m, 2H). ^13^C-NMR: δ 170.65, 166.99, 160.63, 160.18, 158.08, 142.90, 137.62, 130.76, 130.46, 129.27, 128.20, 126.51, 125.53, 118.77, 112.31, 107.44, 55.16, 37.34. ESI-MS (*m*/*z*) = 490.98 [M + H]^+^, HRMS (ESI) calcd. For C_26_H_21_F_2_N_4_O_4_^+^: [M + H]^+^ *m*/*z*: 491.1525, found: 491.1500.

*(S)-4-(2-(5-(3-Chloro-2,6-difluorophenyl)-1H-pyrazole-3-carboxamido)-3-phenylpropanamido)benzoicacid* (**7k**): white solid product (220 mg, 70.2% yield), m.p.: 129–131 °C, decomposition. ^1^H-NMR: δ 13.80 (s, 1H), 12.74 (s, 1H), 10.53 (s, 1H), 7.91–7.89 (d, *J* = 8.4 Hz, 2H), 7.73–7.70 (m, 3H), 7.34–7.04 (m, 8H), 4.93–4.87 (m, 1H), 3.18–3.15 (m, 2H). ^13^C-NMR: δ 171.23, 167.60, 157.27, 143.45, 138.22, 131.08, 129.86, 128.81, 127.13, 126.20, 119.39, 117.07, 113.99, 113.77, 108.30, 55.82, 37.94. ESI-MS (*m*/*z*) = 525.00 [M + H]^+^, HRMS (ESI) calcd. For C_26_H_20_ClF_2_N_4_O_4_^+^: [M + H]^+^ *m*/*z*: 525.1136, found: 525.1116.

*(S)-4-(2-(5-(4-Chloro-2,6-difluorophenyl)-1H-pyrazole-3-carboxamido)-3-phenylpropanamido)benzoic acid* (**7l**): white solid product (220 mg, 70.2% yield), m.p.: 123–125 °C, decomposition. ^1^H-NMR: δ 13.93–13.77 (m, 1H), 12.78–12.72 (m, 1H), 10.59 (s, 1H), 9.04–9.02 (m, 1H), 7.91–7.89 (d, *J* = 8.8 Hz, 2 H), 7.73–7.71 (d, *J* = 8.8 Hz, 2H), 7.54 (s, 2H), 7.36–7.34 (m, 2H), 7.27–7.24 (m, 2H), 7.18–7.10 (m, 1H), 6.99–6.96 (m, 1H), 4.91–4.86 (m, 1H), 3.17–3.14 (m, 2H). ^13^C-NMR: δ 171.30, 167.68, 158.58, 143.52, 138.27, 131.07, 129.91, 128.80, 127.13, 126.19, 119.34, 114.11, 113.83, 108.14, 55.92, 37.86. ESI-MS (*m*/*z*) = 525.01 [M + H]^+^, HRMS (ESI) calcd. For C_26_H_20_ClF_2_N_4_O_4_^+^: [M + H]^+^ *m*/*z*: 525.1136, found: 525.1126.

*(S)-4-(2-(5-(3-Chlorophenyl)-4-methyl-1H-pyrazole-3-carboxamido)-3-phenylpropanamido)benzoic acid* (**7m**): white solid product (29 mg, 9.8% yield), m.p.: 164–166 °C, decomposition. ^1^H-NMR: δ 13.48 (s, 1H), 12.70 (s, 1H), 10.48 (s, 1H), 8.01–7.98 (m, 1H), 7.91–7.89 (m, 2H), 7.71–7.69 (m, 2H), 7.60 (s, 1H), 7.54–7.49 (m, 3H), 7.29–7.21 (m, 4H), 7.21–7.19 (m, 1H), 4.90–4.88 (m, 1H), 3.17–3.15 (m, 2H), 2.29 (s, 3H). ESI-MS (*m*/*z*) = 503.00 [M + H]^+^, HRMS (ESI) calcd. For C_27_H_24_ClN_4_O_4_^+^: [M + H]^+^ *m*/*z*: 503.1481, found: 503.1467.

*(S)-4-(2-(5-(3-Chlorophenyl)-4-ethyl-1H-pyrazole-3-carboxamido)-3-phenylpropanamido)benzoic acid* (**7n**): white solid product (227 mg, 73.5% yield), m.p.: 126–128 °C, decomposition. ^1^H-NMR: δ 13.46 (s, 1H), 12.74 (s, 1H), 10.54 (s, 1H), 8.01 (s, 1H), 7.91–7.89 (d, *J* = 8.4 Hz, 2H), 7.72–7.70 (d, *J* = 8.8 Hz, 2H), 7.54–7.45 (m, 4H), 7.31–7.24 (m, 4H), 7.20–7.16 (m, 1H), 4.93–4.88 (qd, *J* = 8.0 Hz, 1H), 3.21–3.10 (m, 2H), 2.75–2.69 (qd, *J* = 7.2 Hz, 2H), 1.04–1.01 (m, 3H). ESI-MS (*m*/*z*) = 516.91 [M + H]^+^, HRMS (ESI) calcd. For C_28_H_26_ClN_4_O_4_^+^: [M + H]^+^ *m*/*z*: 517.1637, found: 517.1624.

*(S)-Ethyl 5-(2-(3-(3-chloro-2-fluorophenyl)-1H-pyrazole-5-carboxamido)-3-phenylpropanamido)-1H-indole-2-carboxylate* (**15o**): To a mixture of 3-(3-chloro-2-fluorophenyl)-1*H*-pyrazole-5-carboxylic acid (**11f**, 136 mg, 0.57 mmol) in DMF (3 mL) was added 1-hydroxybenzotriazole (139 mg, 1.04 mmol), (*S*)-ethyl 5-(2-amino-3-phenylpropanamido)-1*H*-indole-2-carboxylate hydrochloride (**14a**, 200 mg, 0.52 mmol), *N*-(3-dimethylaminopropyl)-*N*′-ethylcarbodiimide hydrochloride (198 mg, 1.04 mmol) and *N*,*N*-diisopropylethylamine (200 mg, 1.56 mmol), then the reaction mixture was stirred at room temperature overnight.TLC analysis indicated reaction was complete and H_2_O (30 mL) was added. The suspension was stirred for 0.5h and filtered. The filter cake was washed by H_2_O (5 mL) to get crude product **15o** (130 mg, 44.2% yield) as a brown solid, which was used for next step without further purification.

*(S)-5-(2-(3-(3-Chloro-2-fluorophenyl)-1H-pyrazole-5-carboxamido)-3-phenylpropanamido)-1H-indole-2-carboxylic acid* (**7o**): To a suspension of compound **15o** (130 mg, 0.23 mmol) in EtOH (3 mL) and H_2_O (1.5 mL) was added LiOH·H_2_O (38 mg, 0.92 mmol) and the reaction mixture was stirred at 40 °C for 6 h when TLC analysis indicated completion of reaction. The reaction mixture was evaporated on a rotary evaporator in vacuum and H_2_O (4 mL) was added. The solution was extracted by MTBE (2 mL), acidified by hydrochloric acid (1 M) to pH 3–4. After stirred for 0.5 h, the suspension was filtered, washed by H_2_O (5 mL) and the filter cake was dried at 50 °C for 2 h to afford **7o** as a white solid (112 mg, 39.6% yield for 2 steps), m.p.: 172–174 °C, decomposition. ^1^H-NMR: δ 13.84 (s, 1H), 12.81 (s, 1H), 11.67 (s, 1H), 10.11 (s, 1H), 7.98 (s, 1H), 7.88 (s, 1H), 7.58 (s, 1H), 7.52–7.11 (m, 11H), 7.04 (s, 1H), 4.93–4.87 (m, 1H), 3.20–3.10 (m, 2H). HRMS (ESI) calcd. for C_28_H_22_ClFN_5_O_4_^+^: [M + H]^+^ *m*/*z*: 546.1339, found: 546.1325.

Compounds **7p**~**7w** were synthesized according to the procedure described for the preparation of **7o**.

*(S)-5-(2-(3-(3-Chloro-2,6-difluorophenyl)-1H-pyrazole-5-carboxamido)-3-phenylpropanamido)-1H-indole-2-carboxylic acid* (**7p**): white solid product (91 mg, 31.2% yield), m.p.: 164–166 °C, decomposition. ^1^H-NMR: δ 13.96 (s, 1H), 12.98 (s, 1H), 11.61 (s, 1H), 10.30 (s, 1H), 8.93 (s, 1H), 7.96 (s, 1H), 7.85 (s, 1H), 7.71–7.17 (m, 9H), 6.99 (s, 1H), 4.90–4.89 (m, 1H), 3.20–3.16 (m, 2H). ESI-MS (*m*/*z*) = 563.87 [M + H]^+^, HRMS (ESI) calcd. For C_28_H_21_ClF_2_N_5_O_4_^+^: [M+ H]^+^ *m*/*z*: 564.1245, found: 564.1234.

*(S)-5-(2-(3-(3-Cchlorophenyl)-4-methyl-1H-pyrazole-5-carboxamido)-3-phenylpropanamido)-1H-indole-2-carboxylic acid* (**7q**): white solid product (60 mg, 21.5% yield), m.p.: 154–156 °C, decomposition. ^1^H-NMR: δ 13.48 (s, 1H), 11.66 (s, 1H), 10.06 (s, 1H), 7.98 (s, 1H), 7.96–7.91 (m, 1H), 7.60 (s, 1H), 7.52 (s, 1H), 7.38–7.14 (m, 9H), 7.03 (s, 1H), 4.93–4.87 (m, 1H), 3.19–3.16 (m, 2H), 2.31 (s, 3H). ESI-MS (*m*/*z*) = 541.99 [M + H]^+^, HRMS (ESI) calcd. For C_29_H_25_ClN_5_O_4_^+^: [M + H]^+^ *m*/*z*: 542.1590, found: 542.1577.

*(S)-5-(2-(3-(3-Chlorophenyl)-1,4-dimethyl-1H-pyrazole-5-carboxamido)-3-phenylpropanamido)-1H-indole-2-carboxylic acid* (**7r**): white solid product (59 mg, 20.9% yield), m.p.: 148–150 °C, decomposition. ^1^H-NMR: δ 12.86 (s, 1H), 11.66 (s, 1H), 10.05 (s, 1H), 7.94 (s, 1H), 7.85–7.83 (d, *J* = 8.4 Hz, 1H), 7.56–7.53 (m, 3H), 7.40–7.24 (m, 6H), 7.20–7.18 (m, 1H), 7.03 (s, 1H), 4.94–4.89 (m, 1H), 3.75 (s, 3H), 3.18–3.10 (m, 2H), 2.09 (s, 3H). HRMS (ESI) calcd. For C_30_H_27_ClN_5_O_4_^+^: [M + H]^+^ *m*/*z*: 556.1746, found: 556.1735.

*(S)-5-(2-(3-(3-Chloro-2-fluorophenyl)-1H-pyrazole-5-carboxamido)-3-(4-fluorophenyl)propanamido)-1H-indole-2-carboxylic acid* (**7s**): white solid product (151 mg, 54.3% yield), m.p.: 186–188 °C, decomposition. ^1^H-NMR: δ 13.87 (s, 1H), 12.99–12.88 (m, 1H), 11.64 (s, 1H), 10.15 (s, 1H), 8.99–8.91 (m, 1H), 7.97 (s, 1H), 7.94 (s, 1H), 7.58 (s, 1H), 7.39–7.29 (m, 5H), 7.15–7.02 (m, 2H), 6.96 (s, 1H), 4.90–4.85 (m, 1H), 3.30–3.09 (m, 2H). ^13^C-NMR: δ 169.64, 163.18, 162.37, 159.96, 155.40, 152.91, 134.32, 134.16, 131.74, 131.32, 131.24, 130.08, 129.90, 126.99, 126.91, 125.79, 120.96, 118.76, 115.04, 114.83, 112.63, 112.26, 107.16, 106.21, 55.32, 36.93. ESI-MS (*m*/*z*) = 563.82 [M + H]^+^, HRMS (ESI) calcd. For C_28_H_21_ClF_2_N_5_O_4_^+^: [M + H]^+^ *m*/*z*: 564.1245, found: 564.1234.

*(S)-5-(2-(3-(3-Chloro-2-fluorophenyl)-1H-pyrazole-5-carboxamido)-3-(3-fluorophenyl)propanamido)-1H-indole-2-carboxylic acid* (**7t**): white solid product (154 mg, 55.7% yield), m.p.: 174–176 °C, decomposition. ^1^H-NMR: δ 13.92–13.75 (m, 1H), 13.03–12.81 (m, 1H), 11.66 (s, 1H), 10.12 (s, 1H), 7.97 (s, 1H), 7.87 (s, 1H), 7.59–7.56 (m, 1H), 7.54–7.27 (m, 5H), 7.21–6.93 (m, 4H), 4.94–4.88 (m, 1H), 3.29–3.13 (m, 2H). ESI-MS (*m*/*z*) = 563.84 [M + H]^+^, HRMS (ESI) calcd. For C_28_H_21_ClF_2_N_5_O_4_^+^: [M + H]^+^ *m*/*z*: 564.1245, found: 564.1234.

*(S)-5-(2-(3-(3-Chloro-2-fluorophenyl)-1H-pyrazole-5-carboxamido)-3-(pyridin-4-yl)propanamido)-1H-indole-2-carboxylic acid* (**7u**): grey solid product (92 mg, 32.7% yield), m.p.: 199–201 °C, decomposition.^1^H-NMR: δ 13.87 (s, 1H), 12.83 (s, 1H), 11.69 (s, 1H), 8.96 (s, 1H), 8.50 (s, 1H), 7.98(s, 1H), 7.93 (s, 1H), 7.69–7.39 (m, 6H), 7.05 (s, 1H), 5.00–4.95 (m, 1H), 3.27–3.17 (m, 2H). ESI-MS (*m*/*z*) = 547.01 [M + H]^+^, HRMS (ESI) calcd. For C_27_H_21_ClFN_6_O_4_^+^: [M + H]^+^ *m*/*z*: 547.1291, found: 547.1279.

*(S)-5-(2-(3-(3-Chloro-2-fluorophenyl)-1H-pyrazole-5-carboxamido)-3-(pyridin-3-yl)propanamido)-1H-indole-2-carboxylic acid* (**7v**): grey solid product (240 mg, 85.3% yield), m.p.: 175–177 °C, decomposition. ^1^H-NMR: δ 13.86 (s, 1H), 12.88 (s, 1H), 11.69 (s, 1H), 10.15 (s, 1H), 8.99 (s, 1H), 8.57 (s, 1H), 8.41–8.40 (d, *J* = 4.4 Hz, 1H), 7.98 (s, 1H), 7.88(s, 1H), 7.81–7.80 (d, *J* = 6.8 Hz, 1H), 7.52 (s, 1H), 7.39–7.10 (m, 4H), 7.04 (s, 1H), 4.95–4.89 (m, 1H), 3.25–3.14 (m, 2H). ESI-MS (*m*/*z*) = 547.05 [M + H]^+^, HRMS (ESI) calcd. For C_27_H_21_ClFN_6_O_4_^+^: [M + H]^+^ *m*/*z*: 547.1291, found: 547.1272.

*(S)-5-(2-(3-(3-Chloro-2-fluorophenyl)-1H-pyrazole-5-carboxamido)-3-(pyridin-2-yl)propanamido)-1H-indole-2-carboxylic acid* (**7w**): white solid product (109 mg, 38.7% yield), m.p.: 192–194 °C, decomposition. ^1^H-NMR: δ 11.68 (s, 1H), 10.19 (s, 1H), 8.71–8.70 (d, *J* = 4.4 Hz, 1H), 8.12 (s, 1H), 7.97 (s, 1H), 7.89–7.86 (m, 1H,), 7.71–7.70 (d, *J* = 6.0 Hz, 1H), 7.61–7.57 (t, *J* = 6.8 Hz, 2H), 7.36–7.30 (m, 4H), 7.03 (s, 1H), 5.13–5.11 (m, 1H), 3.56–3.39 (m, 2H). ESI-MS (*m*/*z*) = 547.04 [M + H]^+^, HRMS (ESI) calcd. For C_27_H_21_ClFN_6_O_4_^+^: [M + H]^+^ *m*/*z*: 547.1291, found: 547.1275.

*(S)-Ethyl 5-(4-(tert-butoxy)-2-((tert-butoxycarbonyl)amino)-4-oxobutanamido)-1H-indole-2-carboxylate* (**17**): To a mixture of ethyl 5-amino-1*H*-indole-2-carboxylate (2.00 g, 9.8 mmol), pyridine (3.00 g, 19.6 mmol) and (*S*)-4-(*tert*-butoxy)-2-((*tert*-butoxycarbonyl)amino)-4-oxobutanoic acid (**16**, 2.83 g, 9.8 mmol) in CH_2_Cl_2_ (30 mL) was added POCl_3_ (1.16 g, 14.7 mmol) at −10 °C dropwise.After addition, the reaction mixture was stirred at −10 °C for 1 h when TLC analysis indicated completion of reaction. The mixture was washed by hydrochloric acid (1 M, 10 mL), dried by Na_2_SO_4_ and filtered. The filtrate was evaporated in vacuum and the residue was purified on column chromatography (*n*-hexane:ethyl acetate = 50:1 to 2:1) to get **17** as a white solid (1.77 g, 36.7% yield), m.p.: 119–121 °C, decomposition. ^1^H-NMR: δ 11.78 (s, 1H), 9.83 (s, 1H), 7.98 (s, 1H), 7.36 (s, 2H), 7.09–7.08 (m, 1H), 4.45–4.44 (m, 1H), 4.35–4.29 (m, 2H), 2.69–2.65 (m, 1H), 2.52–2.46 (m, 1H), 1.46–1.30 (m, 21H). ESI-MS (*m*/*z*) = 475.90 [M + H]^+^, HRMS (ESI) calcd. For C_24_H_3__3_KN_3_O_7_^+^: [M + K]^+^ *m*/*z*: 514.1950, found: 514.1947.

*(S)-3-((tert-Butoxycarbonyl)amino)-4-((2-(ethoxycarbonyl)-1H-indol-5-yl)amino)-4-oxobutanoic acid* (**18**): To a mixture of **17** (1.7 g, 3.6 mmol) in THF (5 mL) was added TFA (5 mL) at 0 °C. After addition, the mixture was stirred at room temperature for 6 h when TLC analysis indicated completion of reaction. The reaction mixture was evaporated in vacuum, then to the residue was added THF (20 mL), H_2_O (10 mL), Et_3_N (3.6 g, 36 mmol) and (Boc)_2_O (772 mg, 3.6 mmol) and the reaction was stirred at room temperature overnight.TLC analysis showed the reaction was complete and the reaction mixture was evaporated in vacuum. To the residue was added H_2_O (10 mL), extracted by MTBE (3 mL) and acidified by hydrochloric acid (1 M) to pH = 5. The aqueous layer was extracted by ethyl acetate for 3 times (10 mL × 3), and the combined organic layer was dried by Na_2_SO_4_, filtered and concentrated in vacuum to afford **18** as a grey solid (543 mg, 35.8% yield), m.p.: 123–125 °C, decomposition. ^1^H-NMR: δ 11.97 (s, 1H), 11.77 (s, 1H), 9.90 (s, 1H), 7.98 (s, 1H), 7.35 (s, 2H), 7.10–7.08 (m, 1H), 4.42–4.40 (m, 1H), 4.34–4.29 (m, 2H), 2.68–2.63 (m, 1H), 2.56–2.52 (m, 1H), 1.38–1.30 (m, 12H). ESI-MS (*m*/*z*) = 419.82 [M + H]^+^, HRMS (ESI) calcd. For C_20_H_2__5_KN_3_O_7_^+^: [M + K]^+^ *m*/*z*: 458.1324, found: 458.1322.

*(S)-Ethyl 5-(4-(4-acetylpiperazin-1-yl)-2-((tert-butoxycarbonyl)amino)-4-oxobutanamido)-1H-indole-2-carboxylate* (**19a**): To a mixture of **18** (200 mg, 0.48 mmol) in DMF (4 mL) was added 1-(piperazin-1-yl)ethanone (61 mg, 0.48 mmol), *N*-(3-dimethylaminopropyl)-*N*′-ethylcarbodiimide hydrochloride (183 mg, 0.96 mmol), 1-hydroxybenzotriazole (129 mg, 0.96 mmol) and *N*,*N*-diisopropylethylamine (185 mg, 1.43 mmol). After addition, the reaction mixture was stirred at room temperature for 6 h when TLC analysis indicated completion of reaction. Then H_2_O (40 mL) was added and the mixture was extracted by ethyl acetate (15 mL × 3) for 3 times. The combined organic layer was dried, filtered and the filtrate was concentrated in vacuum to get **19a** as a brown solid (176.3 mg, 69.8% yield). ^1^H-NMR: δ 11.76 (s, 1H), 9.81 (s, 1H), 7.94 (s, 1H), 7.36 (s, 1H), 7.09–7.07 (d, *J* = 2.0 Hz, 1H), 6.98–6.96 (d, *J* = 8.0 Hz, 1H), 4.50–4.48 (m, 1H), 4.34–4.29 (m, 2H), 3.45–3.39 (m, 8H), 2.75–2.72 (m, 2H), 1.99 (s, 3H), 1.37–1.30 (m, 12H). ESI-MS (*m*/*z*) = 529.95 [M + H]^+^.

*(S)-Ethyl 5-(2-((tert-butoxycarbonyl)amino)-4-morpholino-4-oxobutanamido)-1H-indole-2-carboxylate* (**19b**): Compounds **19b** were synthesized from 18 and morpholine according to the procedure described for the preparation of **19a**. Grey solid product (137.2 mg, 58.9% yield), m.p.: 144–146 °C, decomposition. ^1^H-NMR: δ 11.76 (s, 1H), 9.81 (s, 1H), 8.00 (s, 1H), 7.36 (s, 2H), 7.08 (s, 1H), 6.96–6.94 (d, *J* = 7.2 Hz, 1H), 4.49–4.48 (m, 1H), 4.34–4.29 (qd, *J* = 7.2, 2H), 3.54–3.41 (m, 8H), 2.72 (m, 2H), 1.38–1.30 (m, 12H). ESI-MS (*m*/*z*) = 488.97 [M + H]^+^, HRMS (ESI) calcd. For C_24_H_3__2_KN_4_O_7_^+^: [M + K]^+^ *m*/*z*: 527.1903, found: 527.1894.

*(S)-Ethyl 5-(4-(4-acetylpiperazin-1-yl)-2-(5-(3-chloro-2-fluorophenyl)-1H-pyrazole-3-carboxamido)-4-oxobutanamido)-1H-indole-2-carboxylate* (**20a**): To a mixture of **19a** (170 mg, 0.32 mmol) in ethyl acetate (1 mL) was added hydrochloric/ethyl acetate (10 mL, saturated solution) and the reaction mixture was stirred at room temperature overnight. Then TLC analysis showed reaction was complete and the suspension was filtered to afford the intermediate. To the intermediate (150 mg, 0.32 mmo) in DMF (3 mL) was added 11f (78 mg, 0.32 mmo), *N*-(3-dimethylaminopropyl)-*N*′-ethylcarbodiimide hydrochloride (124 mg, 0.64 mmol), 1-hydroxybenzotriazole (87 mg, 0.64 mmol), and *N*,*N*-diisopropylethylamine (124 mg, 0.96 mmol) and the reaction mixture was stirred at room temperature for 6 h when TLC analysis indicated completion of reaction. Then H_2_O (30 mL) was added and the suspension was stirred for 10 min, filtered, dried at 50 °C for 4 h to get **20a** as a brown solid (91.2 mg, 43.3% yield), m.p.:156–158 °C, decomposition. ^1^H-NMR: δ 13.94 (m, 1H), 11.78 (s, 1H), 10.04–9.91 (m, 1H), 8.80–8.78 (m, 1H), 8.01 (s, 1H), 7.94–7.82 (m, 1H), 7.62–7.54 (m, 1H), 7.44–7.11 (m, 4H), 7.08 (s, 1H), 5.00 (s, 1H), 4.36–4.29 (m, 2H), 3.58–3.39 (m, 8H), 2.93–2.88 (m, 2H), 1.97 (s, 3H), 1.34–1.30 (m, 3H). ESI-MS (*m*/*z*) = 651.97 [M + H]^+^, HRMS (ESI) calcd. For C_31_H_3__2_ClFN_7_O_6_^+^: [M + H]^+^ *m*/*z*: 652.2081, found: 652.2070.

*(S)-Ethyl 5-(2-(5-(3-chloro-2-fluorophenyl)-1H-pyrazole-3-carboxamido)-4-morpholino-4-oxobutanamido)-1H-indole-2-carboxylate* (**20b**): Compounds **20b** were synthesized from **19b** and **11f** according to the procedure described for the preparation of **20a**. Grey solid product (90.3 mg, 52.2 % yield), m.p.:136–138 °C, decomposition. ^1^H-NMR: δ 13.90 (s, 1H), 11.78 (s, 1H), 9.98 (s, 1H), 8.01 (s, 1H), 7.88 (s, 1H), 7.60–7.53 (m, 2H), 7.41–7.30 (m, 4H), 7.08 (s, 1H), 5.03–4.97 (m, 1H), 4.38–4.29 (m, 2H), 3.63–3.38 (m, 8H), 2.93–2.80 (m, 2H), 1.38–1.30 (m, 3H). ESI-MS (*m*/*z*) = 610.95 [M + H]^+^, HRMS (ESI) calcd. For C_29_H_2__9_ClFN_6_O_6_^+^: [M + H]^+^ *m*/*z*: 611.1806, found: 611.1811.

*(S)-5-(4-(4-Acetylpiperazin-1-yl)-2-(5-(3-chloro-2-fluorophenyl)-1H-pyrazole-3-carboxamido)-4-oxobutanamido)-1H-indole-2-carboxylic acid* (**7x**): To a mixture of **20a** (85 mg, 0.13 mmol) in EtOH (4 mL) and H_2_O (2 mL) was added LiOH·H_2_O (40 mg, 0.95) and the reaction mixture was stirred at room temperature for 5 h when TLC analysis indicated completion of reaction. The mixture was evaporated on a rotary evaporator in vacuum, then the residue was acidified by hydrochloric acid (1 M) to pH 3–4 and filtered. The filter cake was dried at 50 °C for 4 h to get **7x** as a white solid (62 mg, 76.4% yield), m.p.: 101–103 °C, decomposition. ^1^H-NMR (400 MHz, DMSO-d6): δ 13.94 (s, 1H), 11.63 (s, 1H), 10.00 (s, 1H), 7.98 (s, 1H), 7.89 (s, 1H), 7.58 (s, 1H), 7.43–7.32 (m, 4H), 7.01 (s, 1H), 5.01–5.00 (m, 1H), 3.57–3.47 (m, 6H), 2.95–2.85 (m, 4H), 2.00 (s, 3H). ESI-MS (*m*/*z*) = 623.94 [M + H]^+^, HRMS (ESI) calcd. For C_29_H_28_ClFN_7_O_6_^+^: [M + H]^+^ *m*/*z*: 624.1768, found: 624.1755.

*(S)-5-(2-(3-(3-Chloro-2-fluorophenyl)-1H-pyrazole-5-carboxamido)-4-morpholino-4-oxobutanamido)-1H-indole-2-carboxylic acid* (**7y**): Compounds **7y** were synthesized from **20b** according to the procedure described for the preparation of **7x**. White solid product (59 mg, 77.8% yield), m.p.: 175–177 °C, decomposition. ^1^H-NMR: δ 13.95–13.84 (m, 1H), 11.62–11.60 (m, 1H), 10.01–9.98 (m, 1H), 8.81 (s, 1H), 8.38–8.36 (m, 1H), 7.98 (s, 1H), 7.90–7.82 (m, 1H), 7.58 (s, 1H), 7.48–7.28 (m, 3H), 7.00 (s, 1H), 5.01–4.92 (m, 1H), 3.57–3.42 (m, 4H), 3.39–3.29 (m, 4H), 2.96–2.92 (m, 2H). ESI-MS (*m*/*z*) = 582.96 [M + H]^+^, HRMS (ESI) calcd. For C_27_H_26_ClFN_6_O_6_^+^: [M + H]^+^ *m*/*z*: 583.1503, found: 583.1485.

*(S)-2-(5-(3-Chloro-2-fluorophenyl)-1H-pyrazole-3-carboxamido)-3-(4-nitrophenyl)propanoic acid* (**22**): A mixture of 3-(3-chloro-2-fluorophenyl)-1*H*-pyrazole-5-carboxylic acid (**11f**, 2.2 g, 8.43 mmol), (*S*)-methyl 2-amino-3-(4-nitrophenyl)propanoate (**21**, 2.0 g, 8.43 mmol), 1-hydroxybenzotriazole (2.3 g, 17.04 mmol), *N*-(3-dimethylaminopropyl)-*N*′-ethylcarbodiimide hydrochloride (3.2 g, 17.04 mmol) and *N*,*N*-diisopropylethylamine (3.2 g, 24.80 mmol)in DMF (20 mL) was stirred at room temperature overnight. Then TLC analysis showed reaction was complete and H_2_O (200 mL) was added, then the suspension was stirred for 0.5 h andfiltered. The filter cake was transferred to a round-bottomed flask, and MeOH (60 mL), H_2_O (30 mL) and LiOH·H_2_O (1.0 g, 23.8 mmol) was added. The reaction mixture was stirred at room temperature for 2 h when TLC analysis indicated completion of reaction, evaporated on a rotary evaporator in vacuum, then H_2_O (50 mL) was added, acidified by hydrochloric acid (1 M) to pH 3–4 and filtered. The filter cake was dried at 50 °C for 8 h to afford **22** as a yellow solid (3.25 g, 89.0% yield), m.p.: 167–169 °C, decomposition. ^1^H-NMR: δ 14.01–13.79 (m, 1H), 8.11–8.02 (m, 2H), 7.87–7.84 (m, 1H), 7.59–7.56 (m, 1H), 7.49–7.42 (m, 2H), 7.36–7.22 (m, 1H), 7.06 (s, 1H), 4.42–4.29 (m, 1H), 3.32–3.19 (m, 2H). ESI-MS (*m*/*z*) = 432.94 [M + H]^+^, HRMS (ESI) calcd. For C_19_H_1__5_ClFN_4_O_5_^+^: [M + H]^+^ *m*/*z*: 433.0710, found: 433.0708.

*(S)-Ethyl 5-(3-(4-aminophenyl)-2-(5-(3-chloro-2-fluorophenyl)-1H-pyrazole-3-carboxamido)propanamido)-1H-indole-2-carboxylate* (**23**): A mixture of compound **22** (3.00 g, 6.93 mmol), ethyl 5-amino-1*H*-indole-2-carboxylate (1.42 g), *N*-(3-dimethylaminopropyl)-*N*′-ethylcarbodiimide hydrochloride (2.66 g, 13.9 mmol), 1-hydroxybenzotriazole (1.87 g, 13.9 mmol) and *N*,*N*-diisopropylethylamine (1.79 g, 13.9 mmol) in DMF(20 mL) was stirred at room temperature overnight when TLC analysis indicated completion of reaction, then H_2_O (200 mL) was added. The suspension was stirred for 10 min and filtered. The filter cake was dried at room temperature overnight and resolve in MeOH (200 mL) and ethyl acetate (100 mL). To the solution was added Pd/C (10%, 0.30 g) and the reaction mixture was stirred at the atmosphere of H_2_overnight when TLC analysis indicated completion of reaction. The suspension was filtered and the filtrate was concentrated in vacuum to afford intermediate **23** as a grey solid (3.1 g, 75.6% yield), m.p.: 120–122 °C, decomposition. ^1^H-NMR: δ 13.77 (s, 1H), 11.80 (s, 1H), 10.20 (s, 1H), 8.99–8.69 (m, 2H), 7.99 (s, 1H), 7.91 (s, 1H), 7.38–6.98 (m, 7H), 6.91 (s, 1H), 6.81–6.80 (d, *J* = 2.4 Hz, 2H), 4.88–4.86 (m, 1H), 4.34–4.29 (qd, *J* = 6.8 Hz, 2H), 3.17–3.10 (m, 2H), 1.34–1.31 (m, 3H). ^13^C-NMR: δ 170.51, 161.92, 147.33, 135.04, 132.57, 130.40, 128.58, 127.48, 127.26, 126.30, 125.51, 119.84, 114.71, 113.25, 112.79, 108.33, 106.65, 61.05, 54.03, 40.76, 14.94. ESI-MS (*m*/*z*) = 588.98 [M + H]^+^, HRMS (ESI) calcd. For C_30_H_2__7_ClFN_6_O_4_^+^: [M + H]^+^ *m*/*z*: 589.1688, found: 589.1755.

*(S)-5-(3-(4-Aminophenyl)-2-(5-(3-chloro-2-fluorophenyl)-1H-pyrazole-3-carboxamido)propanamido)-1H-indole-2-carboxylic acid* (**7z**): To a mixture of compound **23** (140 mg, 0.24 mmol) in MeOH (4 mL) and H_2_O (2 mL) was added LiOH·H_2_O (52 mg, 1.20 mmol) and the mixture was stirred at 40 °C for 4 h when TLC analysis indicated completion of reaction. The mixture was concentrated and H_2_O (2 mL) was added. The solution was acidified with hydrochloric acid (1 M) to pH = 5, filtered and the filter cake was washed by H_2_O (2 mL), dried at 50 °C for 3 h to afford **7z** as a brown solid (98.0 mg, 72.8% yield), m.p.: 209–211 °C, decomposition. ^1^H-NMR: δ 13.88–13.86 (m, 1H), 11.64 (s, 1H), 10.10–10.04 (m, 1H), 8.82 (s, 1H), 7.96 (s, 1H), 7.89 (s, 1H), 7.58 (s, 1H), 7.57–7.29 (m, 6H), 7.22–6.98 (m, 3H), 6.45–6.43 (d, *J* = 8.0 Hz, 2H), 4.80–4.74 (m, 1H), 3.02–2.92 (m, 2H). ESI-MS (*m*/*z*) = 560.92 [M + H]^+^, HRMS (ESI) calcd. For C_28_H_23_ClFN_6_O_4_^+^: [M + H]^+^ *m*/*z*: 561.1448, found: 561.1439.

*Ethyl 5-((2S)-2-(5-(3-chloro-2-fluorophenyl)-1H-pyrazole-3-carboxamido)-3-(4-(2-methylcyclopropane-carboxamido)phenyl)propanamido)-1H-indole-2-carboxylate* (**24za**): To a mixture of compound **23** (200 mg, 0.34 mmol), 2-methylcyclopropanecarboxylic acid (34 mg, 0.34 mmol) and pyridine (54 mg, 0.68 mmol) in CH_2_Cl_2_ (5 mL) was added POCl_3_(78 mg, 0.51 mmol) at −10 °C dropwise. After addition, the reaction mixture was stirred at −10 °C for 1 h when TLC analysis indicated completion of reaction. The mixture was added H_2_O (0.5 mL), concentrated in vacuum, and the residue was purified by Prep-TLC to afford desired product **24za** as a brown solid (76 mg, 33.3% yield), m.p.: 227–229 °C, decomposition. ^1^H-NMR: δ 13.85 (s, 1H), 11.80 (s, 1H), 10.10 (s, 1H), 9.99 (s, 1H), 7.98 (s, 1H), 7.91 (s, 1H), 7.57 (s, 1H), 7.48–7.10 (m, 9H), 7.05 (s, 1H), 4.86–4.85 (m, 1H), 4.35–4.29 (qd, *J* = 6.8 Hz, 2H), 3.12–3.02 (m, 2H), 1.46–1.45 (m, 1H), 1.34–1.31 (m, 3H), 1.22–1.16 (m, 1H), 1.08–1.05 (m, 3H), 0.96–0.94(m, 1H), 0.58(s, 1H). ESI-MS (*m*/*z*) = 670.92 [M + H]^+^, HRMS (ESI) calcd. For C_35_H_3__3_ClFN_6_O_5_^+^: [M + H]^+^ *m*/*z*: 671.2180, found: 671.2178.

*5-((2S)-2-(5-(3-Chloro-2-fluorophenyl)-1H-pyrazole-3-carboxamido)-3-(4-(2-methylcyclopropane-carboxamido)phenyl)propanamido)-1H-indole-2-carboxylic acid* (**7za**): Compounds **7za** was synthesized from compound **24za** according to the procedure described for the preparation of **7z**. Grey solid product (45 mg, 63.7% yield), m.p.: 212–214 °C, decomposition. ^1^H-NMR: δ 13.85 (s, 1H), 12.83 (s, 1H), 11.67 (s, 1H), 10.12 (s, 1H), 8.12 (s, 1H), 7.98 (s, 1H), 7.60–7.57 (m, 1H), 7.52–7.04 (m, 6H), 7.03 (s, 1H), 4.88–4.70 (m, 1H), 3.14–3.01 (m, 2H), 1.48–1.44 (m, 1H), 1.21–1.17(m, 1H), 1.16–1.08 (m, 3H), 1.06–1.04 (m, 1H), 0.56–0.59 (m, 1H). ^13^C-NMR: δ 171.23, 169.68, 162.96, 155.35, 152.85, 138.12, 137.95, 134.30, 132.17, 131.71, 130.05, 129.56, 126.92, 125.71, 120.90, 120.73, 118.80, 112.57, 112.21, 107.24, 106.11, 55.26, 37.26, 23.07, 17.61, 15.21, 11.90. ESI-MS (*m*/*z*) = 642.92 [M + H]^+^, HRMS (ESI) calcd. For C_33_H_29_ClFN_6_O_5_^+^: [M + H]^+^ *m*/*z*: 643.1867, found: 643.1848.

*(S)-Ethyl 5-(2-(5-(3-chloro-2-fluorophenyl)-1H-pyrazole-3-carboxamido)-3-(4-(4-methylpiperazine-1-carboxamido)phenyl)propanamido)-1H-indole-2-carboxylate* (**24zb**): A mixture of compound **23** (300 mg, 0.51 mmol), pyridine (403 mg, 5.1 mmol), 4-methylpiperazine-1-carbonyl chloride hydrochloride (166 mg, 1.02 mmol), and 4-dimethylaminopyridine (13 mg, 0.10 mmol) in DMF (5 mL) was stirred at room temperature overnight when TLC analysis indicated completion of reaction. The mixture was added H_2_O (40 mL) and extracted by ethyl acetate for 3 times (15 mL × 3). The combined organic layer was dried and concentrated in vacuum to get intermediate **24zb** (223 mg, 60.8% yield), m.p.: 174–176 °C, decomposition. ^1^H-NMR: δ 13.86 (s, 1H), 11.80 (s, 1H), 10.14 (s, 1H), 8.44 (s, 1H), 7.99–7.89 (m, 2H), 7.58 (s, 1H), 7.49–7.34 (m, 6H), 7.32–7.21 (m, 2H), 7.09 (s, 1H), 4.86–4.84 (m, 1H), 4.34–4.29 (qd, *J* = 7.2 Hz, 2H), 3.60–3.04 (m, 10H), 2.29 (s, 3H), 1.34–1.31 (m, 3H,). ESI-MS (*m*/*z*) = 715.22 [M + H]^+^, HRMS (ESI) calcd. For C_36_H_3__7_ClFN_8_O_5_^+^: [M + H]^+^ *m*/*z*: 715.2554, found: 715.2543.

*(S)-5-(2-(5-(3-Chloro-2-fluorophenyl)-1H-pyrazole-3-carboxamido)-3-(4-(4-methylpiperazine-1-carboxamido)phenyl)propanamido)-1H-indole-2-carboxylic acid* (**7zb**): sCompound **7zb** was synthesized from compound **24zb** according to the procedure described for the preparation of **7z**. Grey solid product (153 mg, 73.4% yield), m.p.: 203–205 °C, decomposition. ^1^H-NMR: δ 13.88 (s, 1H), 11.66 (s, 1H), 10.10 (s, 1H), 8.44 (s, 1H), 7.97 (s, 1H), 7.88 (s, 1H), 7.58 (s, 1H), 7.38–7.20 (m, 9H), 7.03 (s, 1H), 4.86–4.85 (m, 1H), 3.48–3.44 (m, 6H), 3.13–3.02 (m, 4H), 2.30 (3H, s). ESI-MS (*m*/*z*) = 687.11 [M + H]^+^, HRMS (ESI) calcd. For C_34_H_33_ClFN_8_O_5_^+^: [M + H]^+^ *m*/*z*: 687.2241, found: 687.2222.

*(S)-Ethyl 5-(2-(5-(3-chloro-2-fluorophenyl)-1H-pyrazole-3-carboxamido)-3-(4-(3-oxomorpholino)phenyl)-propanamido)-1H-indole-2-carboxylate* (**24zc**): A mixture of 2-(2-chloroethoxy)acetic acid (48 mg, 0.34 mmol), compound **23** (200 mg, 0.34 mmol) and *N*-ethoxycarbonyl-2-ethoxy-1,2-dihydroquinoline (100 mg, 0.41 mmol) in THF (4 mL) was stirred at 60 °C for 4 h when TLC analysis indicated completion of reaction. The reaction mixture was concentrated in vacuum and THF (5 mL) was added to the residue. To the stirred mixture was added NaH (40 mg, 1 mmol) in portions at room temperature. After addition, the mixture was stirred at room temperature for 5 h and TLC showed reaction was complete. Then NH_4_Cl solution (0.2 mL, concentrated solution) was added and the mixture was concentrated in vacuum. The residue was recrystallized fromn-hexane and ethyl acetate to give **24zc** as a brown solid (174 mg, 76.0% yield). ^1^H-NMR: δ 11.84 (s, 1H), 10.45–10.39 (m, 1H), 8.47 (s, 3H), 8.02 (s, 2H), 7.41–7.39 (m, 3H), 7.28–7.26 (m, 3H), 7.23–7.19 (m, 2H), 4.90 (m, 1H), 4.34–4.28 (qd, *J* = 7.2 Hz, 2H), 3.92–3.89 (m, 2H), 3.68–3.59 (m, 2H), 3.40–3.38 (m, 2H), 3.17–3.07 (m, 2H), 1.33–1.30 (t, *J* = 7.2 Hz, 3H). ESI-MS (*m*/*z*) = 695.17 [M + Na]^+^.

*(S)-5-(2-(5-(3-Chloro-2-fluorophenyl)-1H-pyrazole-3-carboxamido)-3-(4-(3-oxomorpholino)phenyl)-propanamido)-1H-indole-2-carboxylic acid* (**7zc**): Compounds **7zc** was synthesized from compound **24zc** according to the procedure described for the preparation of **7z**. Grey solid product (78.0 mg, 50.4% yield), m.p.: 200–202 °C, decomposition. ^1^H-NMR: δ 13.89 (s, 1H), 11.18 (s, 1H), 10.14 (s, 1H), 7.92–7.88 (m, 2H), 7.56–7.41 (m, 2H), 7.39–7.25 (m, 6H), 4.90–4.89 (m, 1H), 4.15 (s, 2H), 3.93–3.90 (m, 2H), 3.68–3.66 (m, 2H), 3.57–3.55 (m, 2H). ESI-MS (*m*/*z*) = 644.90 [M + H]^+^, HRMS (ESI) calcd. For C_32_H_27_ClFN_6_O_6_^+^: [M + H]^+^ *m*/*z*: 645.1659, found: 645.1637.

### 3.3. Inhibition of FXIa In Vitro

The inhibition of FXIa in vitro was measured using human FXIa (Haematologic Technologies, Essex Junction, VT, USA) and Activated Protein C Chromogenic substrate BIOPHEN CS-21(66) (HYPHEN BioMed, NEUVILLE-SUR-OISE, France) in 96-well microtiter plates at 37 °C. The target compounds and compound **4** was dissolved, diluted in DMSO and analyzed at a final concentration range of 6.5 nM to 84.5 µM respectively. To the 96-well microtiter plates was added 15 µL test compound, 15 µL FXIa (37.5 pM) and 100 µL Tris buffer (adjust to pH 7.4 with hydrochloric acid containing 0.3 M NaCl and 50 mM Tris) in turn respectively. The negative controlwas composed of the same mixed solutions except replacing test compound with DMSO. The positivecontrol was composed of the same mixed solutions except replacing test compound with compound **4**. After incubated for 5 min at 37 °C, Activated Protein C Chromogenic substrate (30 µL, 435 µM) was added and the mixture was incubated at 37 °C. The FXIa inhibitory activity was measured at 405 nm using a Spectra Max M5 (Molecular Devices, Sunnyvale, CA, USA). The **IC_50_** was calculated by IBM SPSS Statistics 22.0 (IBM Inc., North Castle, NY, USA) and the Probit function in it. **IC_50_** values were converted to **Ki** values by the followingrelationship:

Ki = IC_50_/(1 + [S]/Km)

### 3.4. aPTT In Vitro Coagulation Assays

A commercially available automatic Coagulation analyzer (Steellex Science Instrument Co., Ltd., Beijing, China) was employed to measure aPTT. The clotting times were also measured using the instrument itself, in accordance with the manufacturer’s instructions. Increasing concentrations of inhibitor or solvent were added to rabbit (Beijing Longan Experimental Animal Breeding Center, SCXK(Jing)2016-0006, Beijing, China) plasma and incubated for 3 min at 37 °C. APTT was determined by automatic Coagulation analyzer.

### 3.5. Molecular Docking Method

The structure of FXIa receptor was taken from Protein Data Bank with the ID code 5E2O. The receptor was processed using Protein Preparation Wizard, which included solvent deletion, hydrogen addition, bond order assignment and disulfide treatment. The original ligand 5JM was used as the docking center to generate the receptor grid file with a box size of 15 Å. The designed compounds were prepared using LigPrep module, and Epik method was used to determine the possible ionization state at pH = 7.0 ± 2.0 with OPLS-2005 force field. Molecular docking calculations were carried out using Glide with default parameters at standard precision. All these calculations were performed in Schrödinger 2009 software (Glide version 5.5, Schrödinger, New York, NY, USA).

## 4. Conclusions

In summary, we defined 5-phenyl-1*H*-pyrazole-3-carboxylic acid derivatives as privileged fragments and assembled them into **7a**–**7zc** as pharmacophore for FXIa inhibitors. We synthesized and assessed the FXIa inhibitory potency of a series of 5-phenyl-1*H*-pyrazole-3-carboxamide derivatives. Finally, the SAR of them was systemically investigated to afford the lead compound **7za** (FXIa Ki = 90.37 nM, 1.5× aPTT in rabbit plasma = 43.33 μM) which exhibited good in vitro inhibitory potency against FXIa and excellent in vitro coagulation activity and lead optimization is ongoing in our lab. Furthermore, the binding mode of **7za** with FXIawas studied and the results suggest that the 2-methylcyclopropanecarboxamide group of **7za** forms twodirect hydrogen bonds with Tyr58B and Thr35 in the FXIa backbone, making **7za** bind to FXIa in a highly efficient manner.

## Figures and Tables

**Table 1:**
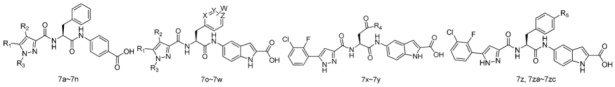
Results of in vitro inhibitory assay of **7a**–**7zc** against FXIa (Ki).

Compound	R_1_	R_2_	R_3_	X, Y, Z	W	R_4_ or R_5_	Ki ^a^
**7a**	3-chlorophenyl	H	H	--	--	--	46.66 μM
**7b**	phenyl	H	H	--	--	--	>84.46 μM
**7c**	2-fluorophenyl	H	H	--	--	--	>84.46 μM
**7d**	2-chlorophenyl	H	H	--	--	--	>84.46 μM
**7e**	4-chlorophenyl	H	H	--	--	--	>84.46 μM
**7f**	3-chloro-2-fluorophenyl	H	H	--	--	--	29.30 μM
**7g**	4-chloro-2-fluorophenyl	H	H	--	--	--	>84.46 μM
**7h**	5-chloro-2-fluorophenyl	H	H	--	--	--	>84.46 μM
**7i**	6-chloro-2-fluorophenyl	H	H	--	--	--	>84.46 μM
**7j**	2,6-difluorophenyl	H	H	--	--	--	>84.46 μM
**7k**	3-chloro-2,6-difluorophenyl	H	H	--	--	--	28.88 μM
**7l**	4-chloro-2,6-difluorophenyl	H	H	--	--	--	>84.46 μM
**7m**	3-chlorophenyl	Me	H	--	--	--	37.15 μM
**7n**	3-chlorophenyl	Et	H	--	--	--	>84.46 μM
**7o**	3-chloro-2-fluorophenyl	H	H	C,C,C	--	--	1.97μM
**7p**	3-chloro-2,6-difluorophenyl	H	H	C,C,C	--	--	8.40 μM
**7q**	3-chlorophenyl	Me	H	C,C,C	--	--	8.94 μM
**7r**	3-chlorophenyl	Me	Me	C,C,C	--	--	11.43 μM
**7s**	3-chloro-2-fluorophenyl	H	H	C,C,C	4-F	--	930.49 nM
**7t**	3-chloro-2-fluorophenyl	H	H	C,C,C	3-F	--	1.66 μM
**7u**	3-chloro-2-fluorophenyl	H	H	C,C,N	--	--	13.27 μM
**7v**	3-chloro-2-fluorophenyl	H	H	C,N,C	--	--	3.52 μM
**7w**	3-chloro-2-fluorophenyl	H	H	N,C,C	--	--	8.99 μM
**7x**	--	--	--	--	--	R_4_: 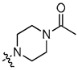	81.18 μM
**7y**	--	--	--	--	--	R_4_: 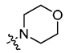	64.01 μM
**7z**	--	--	--	--	--	R_5_: -NH_2_	4.10 μM
**7za**	--	--	--	--	--	R_5_: 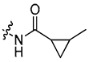	90.37 nM
**7zb**	--	--	--	--	--	R_5_: 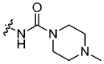	1.94 μM
**7zc**	--	--	--	--	--	R_5_: 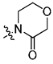	4.82 μM
**4 ^b^**	--	--	--	--	--	--	23.48 nM

^a^ Ki = IC_50_/(1 + [S]/Km); [S]: 435 μM, Km: 395 μM, Ki = IC_50_/1.11; ^b^ Reported value for compound 4: Ki = 3.0 nM against FXIa [18].

**Table 2:** The anticoagulant activity of **7za**.

Compound	1.5× aPTT (μM) (Rabbit)
**7za**	43.33
**4**	3.79
